# Supplementary material for: Planar chlorination engineering induced symmetry-broken single-atom site catalyst for enhanced CO2 electroreduction
Source: Nat Commun. 2025 Feb 15;16:1652. doi: 10.1038/s41467-025-56271-5 (PMC11829013; doi:10.1038/s41467-025-56271-5)
Supplement: Supplementary file 1 — Supplementary Information [file 41467_2025_56271_MOESM1_ESM.pdf]

Planar chlorination engineering induced symmetry-broken single-atom site catalyst for enhanced CO_2 electroreduction

Shengjie Wei[1, 2], Jiexin Zhu[3, 4*], Xingbao Chen[4], Rongyan Yang[5], Kailong Gu[3], Lei Li[6*], Ching-Yu Chiang[7*], Liqiang Mai[4*], Shenghua Chen[3*]

[1]Center Excellence for Environmental Safety and Biological Effects, Beijing Key Laboratory for Green Catalysis and Separation, Department of Chemistry, College of Chemistry and Life Science, Beijing University of Technology, Beijing, 100124, China.

[2]School of Materials Science and Engineering, Nankai University, Tianjin 300350, P. R. China.

[3]National Innovation Platform (Center) for Industry-Education Integration of Energy Storage Technology, School of Chemical Engineering and Technology, Xi'an Jiaotong University, Xi'an, 710049, P. R. China.

[4]State Key Laboratory of Advanced Technology for Materials Synthesis and Processing, Wuhan University of Technology, Wuhan 430070 Hubei, P. R. China.

[5]Key Laboratory of Pollution Processes and Environmental Criteria of Ministry of Education, Tianjin Key Laboratory of Environmental Remediation and Pollution Control, College of Environmental Science and Engineering of Nankai University, Tianjin 300350, P. R. China.

[6]Hefei National Research Center for Physical Sciences at the Microscale, University of Science and Technology of China, Hefei, Anhui 230026, P. R. China.

[7]National Synchrotron Radiation Research Center, Hsinchu 30076, Taiwan.

email:

jxzhu@whut.edu.cn (J.Z.)

leili@mail.ustc.edu.cn (L.L)

chiang.cy@nsrrc.org.tw (C.-Y.C.)

mlq518@whut.edu.cn (L.M.)

shenghchen@xjtu.edu.cn (S.C.)

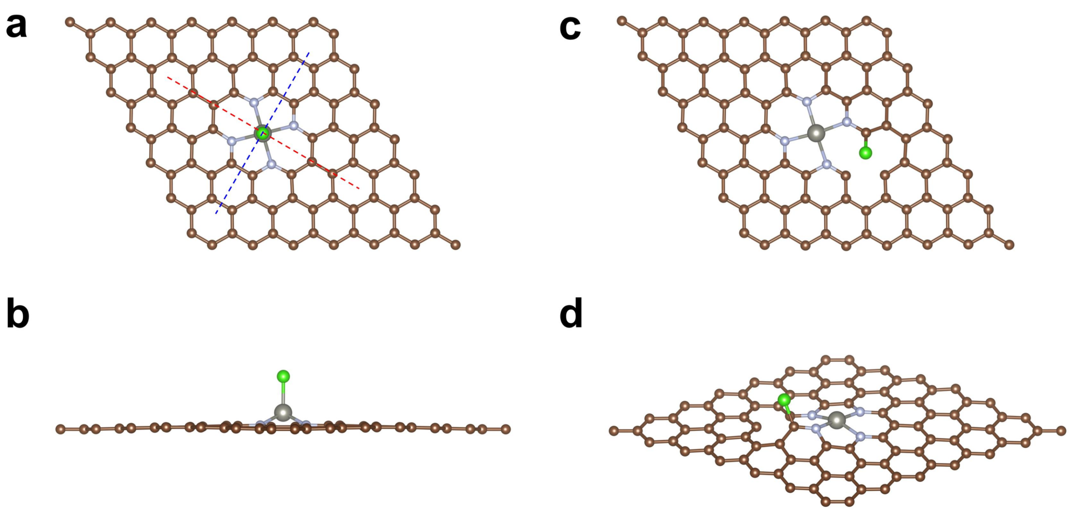

Supplementary Fig. 1. a-b, The scheme of axial chlorination engineering from top view and side view, respectively. **c-d**, The scheme of planar chlorination engineering from top view and side view, respectively. The brown, light blue, dark gray and green balls represented the C, N, Zn and Cl atom, respectively.

As shown in **Supplementary Fig. 1a**, the axial chlorination engineering is introducing the axial Cl coordinating atom on metal-N_4 site, maintaining two planes of symmetry along the metal-Cl bond with C_{2v} symmetry, which does not thoroughly break the geometric symmetry of bonding and electronic distribution. Besides, as shown in **Supplementary Fig. 1b**, after introducing the Cl coordinating atom on metal-N_4 site, the metal atom is dragged out of the planar N-doped graphene, which limits the axial adsorption of reactants on the other side of metal-Cl bond.

As shown in **Supplementary Figs. 1c** and **1d**, the planar chlorination engineering is introducing the C-Cl bond adjacent to metal-N_4 site without existence of any symmetry element, which thoroughly break the planar-like D_{4h} symmetry of metal-N_4 site.

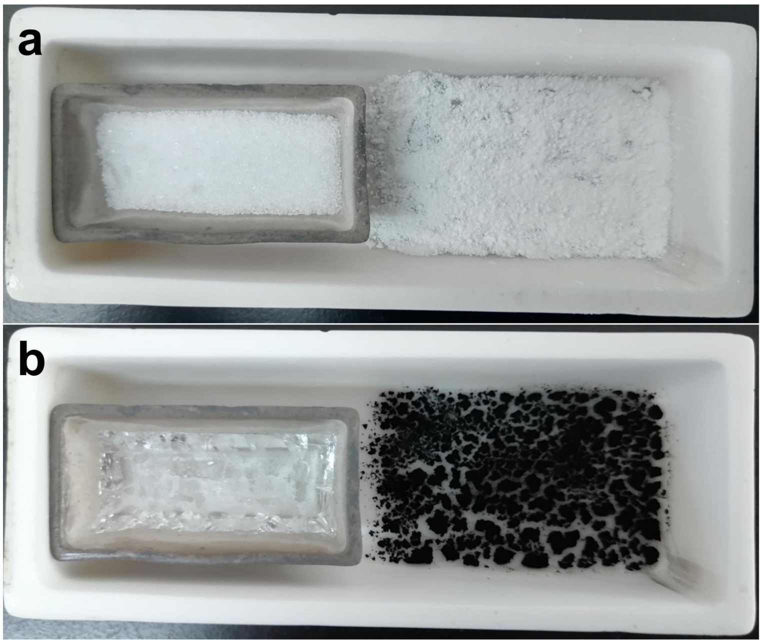

Supplementary Fig. 2. The photographs during the synthetic procedure of the Zn-SA/CNCl-1000 catalyst. **a**, 3 g NaCl in a smaller ceramic boat and 150 mg ZIF-8 powder were put together in a larger ceramic boat with a lid. **b**, After pyrolysis at 1000°C (heating rate: 5°C/min) for 3 h under flowing argon gas (99.999%), the black Zn-SA/CNCl-1000 powder was obtained.

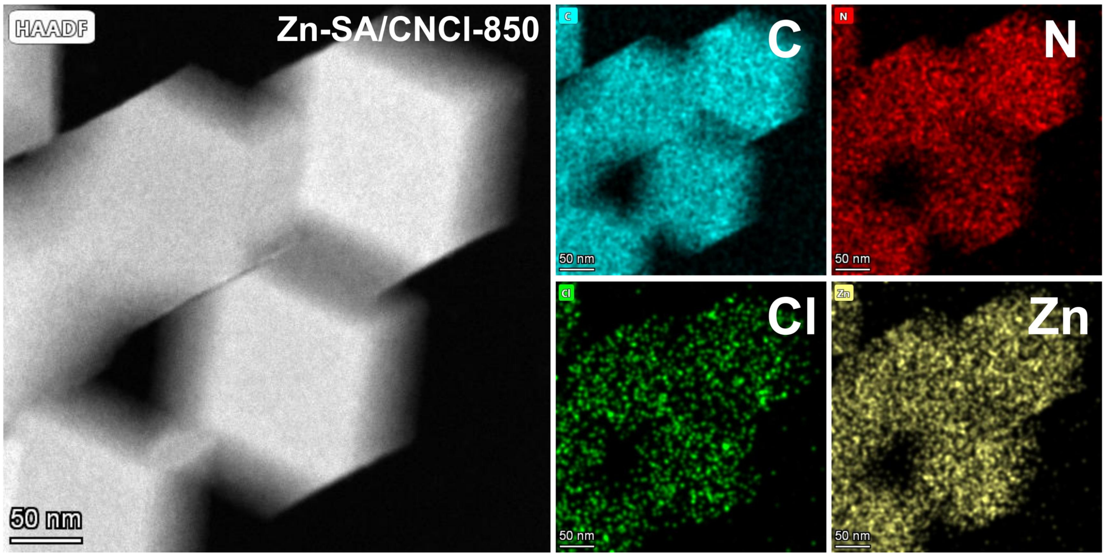

Supplementary Fig. 3. The HAADF-STEM image of Zn-SA/CNCl-850 and the corresponding EDX spectroscopy elemental mapping results.

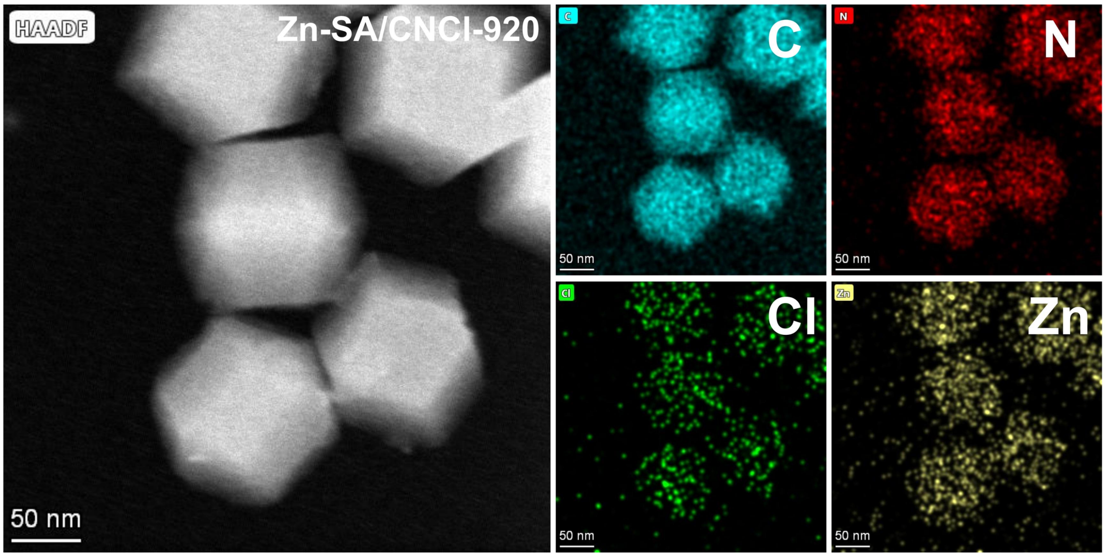

Supplementary Fig. 4. The HAADF-STEM image of Zn-SA/CNCl-920 and the corresponding EDX spectroscopy elemental mapping results.

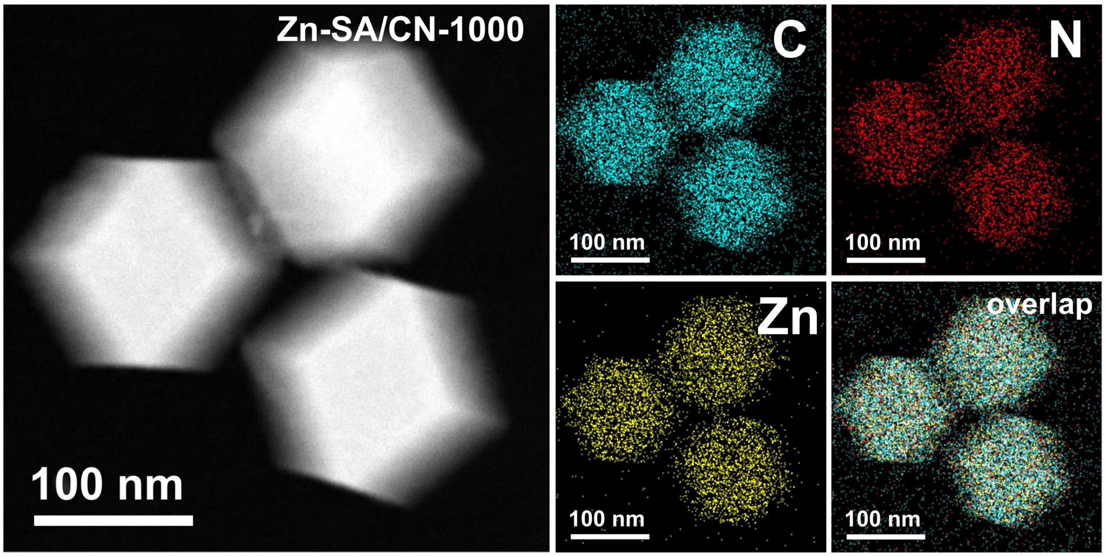

Supplementary Fig. 5. The HAADF-STEM image of Zn-SA/CN-1000 and the corresponding EDX spectroscopy elemental mapping results.

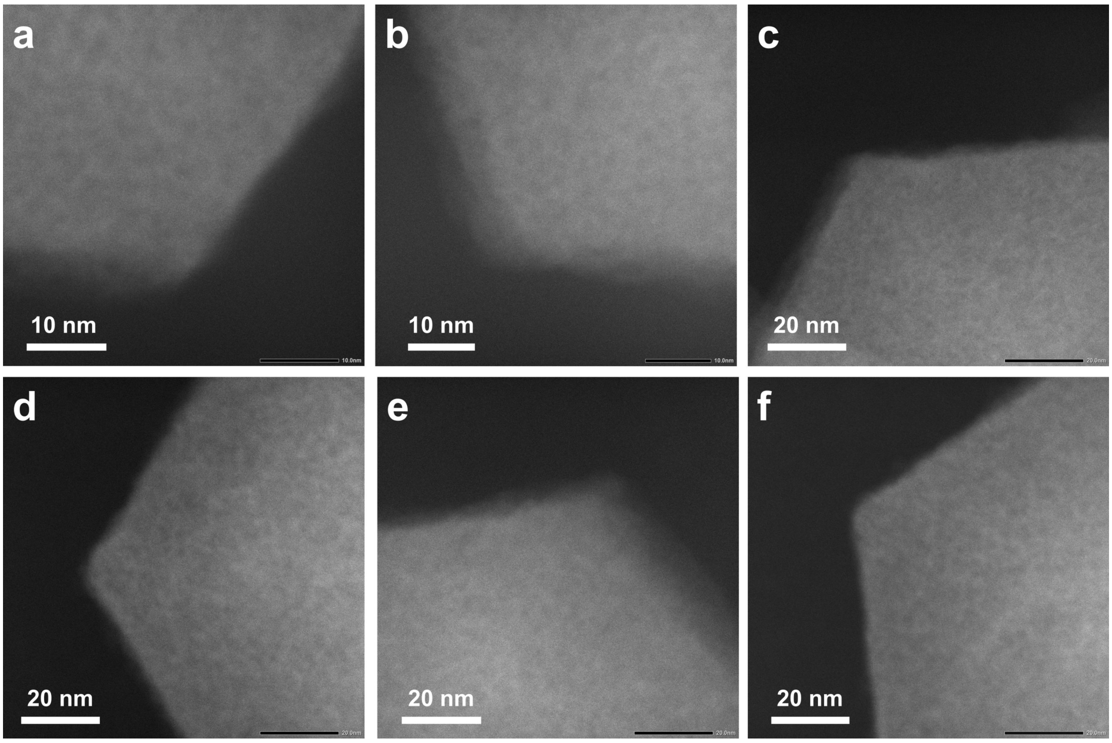

Supplementary Fig. 6. The higher resolution STEM images of Zn-SA/CN-1000. No Zn-based nanoparticles were found in Zn-SA/CN-1000.

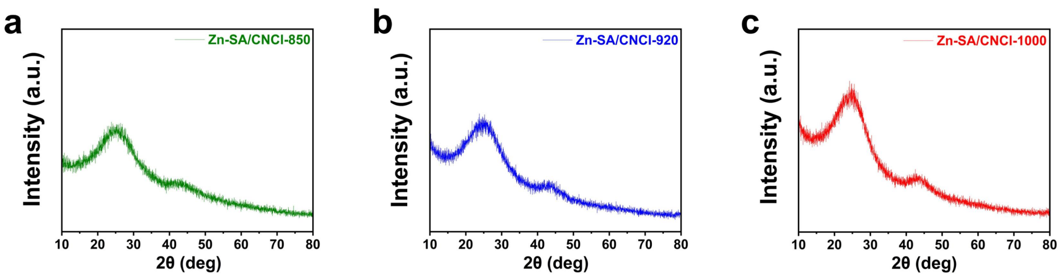

Supplementary Fig. 7. a, The XRD pattern of Zn-SA/CNCl-850. **b**, The XRD pattern of Zn-SA/CNCl-920. **c**, The XRD pattern of Zn-SA/CNCl-1000.

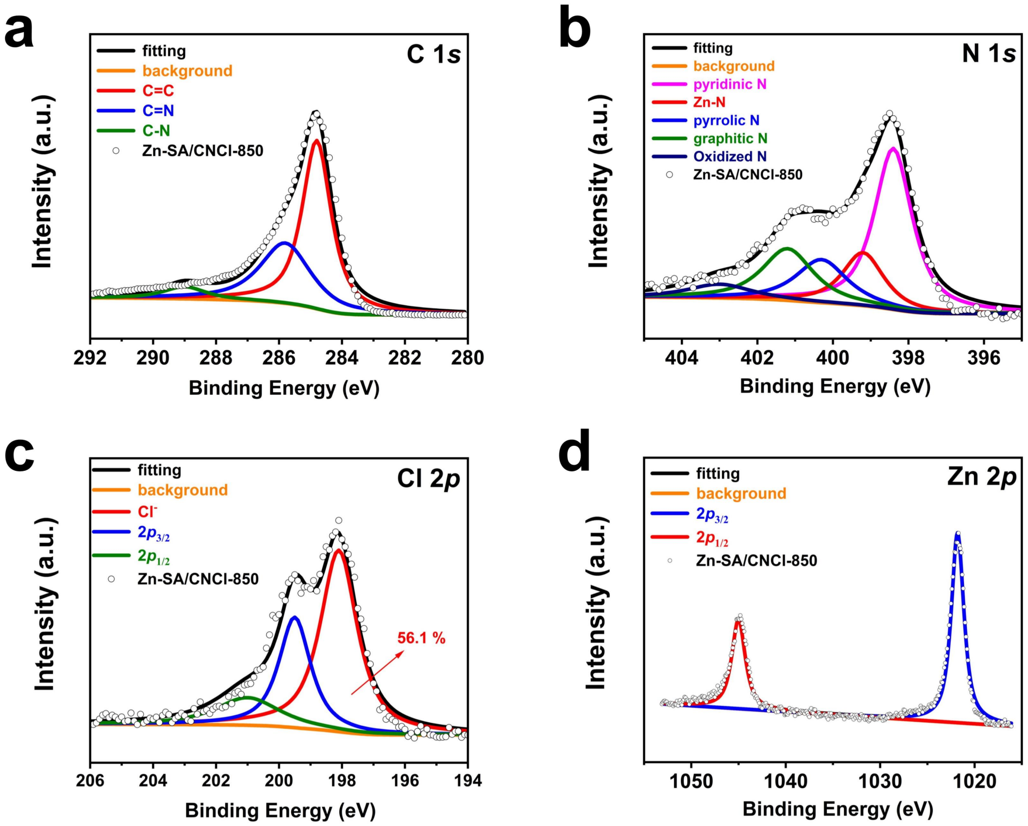

Supplementary Fig. 8. The XPS spectra of Zn-SA/CNCl-850. **a**, XPS spectrum for the C 1*s*. **b**, XPS spectrum for the N 1*s*. **c**, XPS spectrum for the Cl 2*p*. **d**, XPS spectrum for the Zn 2*p*.

The C=C, C=N and C-N bonds (*Angew. Chem. Int. Ed.* **59**, 22465-22469 (2020).) co-existed in the C 1*s* spectrum.

The pyridinic N, Zn-N, pyrrolic N, graphitic N and oxidized N species (*Angew. Chem. Int. Ed.* **60**, 9078-9085 (2021).) co-existed in the N 1*s* spectrum.

The covalent Cl from C-Cl bond and ionic Cl (*Angew. Chem. Int. Ed.* **60**, 27324-27329 (2021).) co-existed in the Cl 2*p* spectrum.

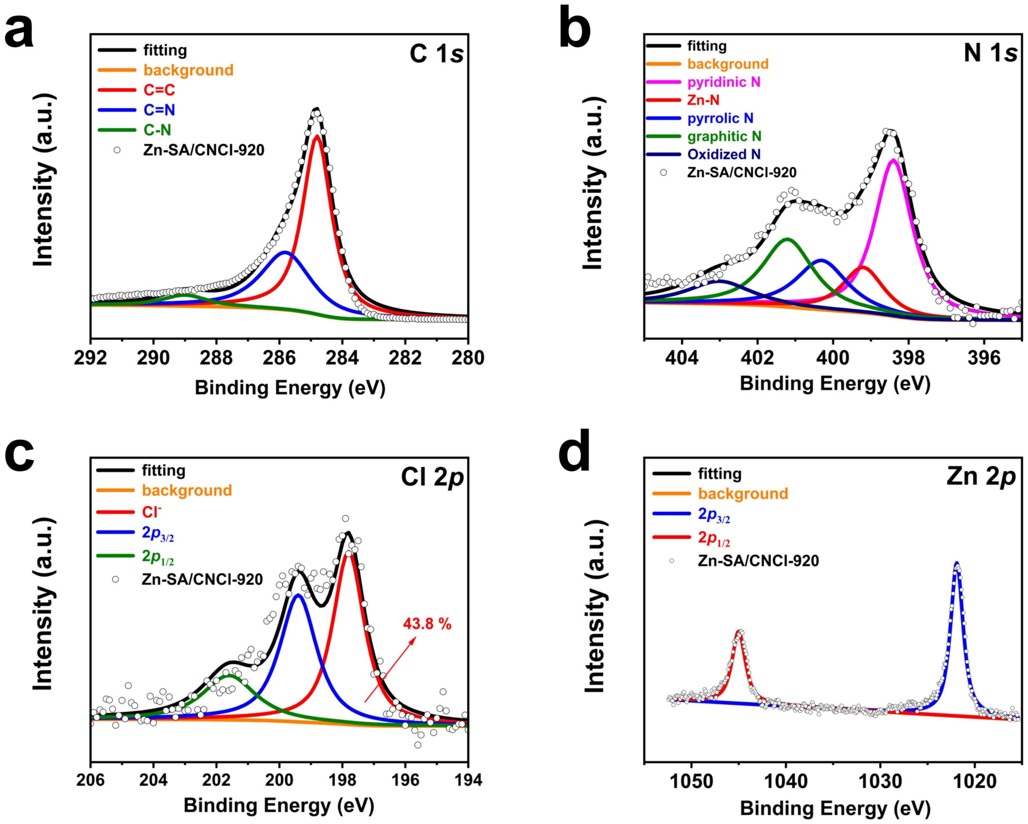

Supplementary Fig. 9. The XPS spectra of Zn-SA/CNCl-920. **a**, XPS spectrum for the C 1*s*. **b**, XPS spectrum for the N 1*s*. **c**, XPS spectrum for the Cl 2*p*. **d**, XPS spectrum for the Zn 2*p*.

The C=C, C=N and C-N bonds (*Angew. Chem. Int. Ed.* **59**, 22465-22469 (2020).) co-existed in the C 1*s* spectrum.

The pyridinic N, Zn-N, pyrrolic N, graphitic N and oxidized N species (*Angew. Chem. Int. Ed.* **60**, 9078-9085 (2021).) co-existed in the N 1*s* spectrum.

The covalent Cl from C-Cl bond and ionic Cl (*Angew. Chem. Int. Ed.* **60**, 27324-27329 (2021).) co-existed in the Cl 2*p* spectrum.

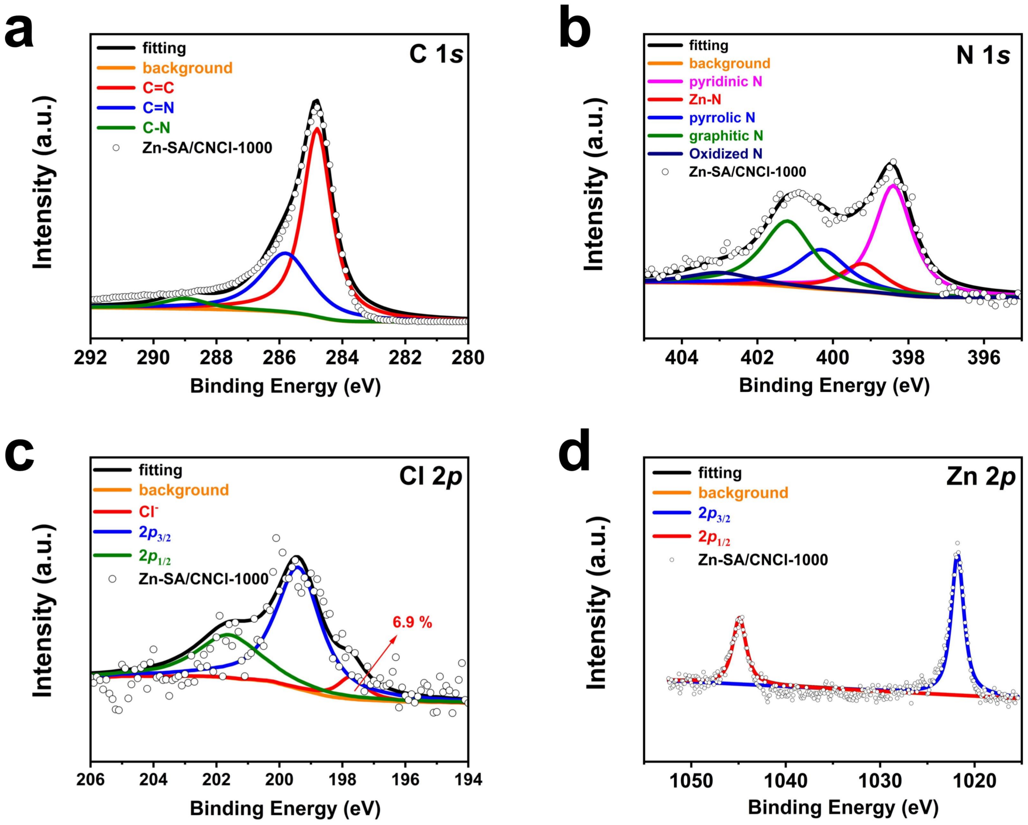

Supplementary Fig. 10. The XPS spectra of Zn-SA/CNCl-1000. **a**, XPS spectrum for the C 1*s*. **b**, XPS spectrum for the N 1*s*. **c**, XPS spectrum for the Cl 2*p*. **d**, XPS spectrum for the Zn 2*p*.

The C=C, C=N and C-N bonds (*Angew. Chem. Int. Ed.* **59**, 22465-22469 (2020).) co-existed in the C 1*s* spectrum.

The pyridinic N, Zn-N, pyrrolic N, graphitic N and oxidized N species (*Angew. Chem. Int. Ed.* **60**, 9078-9085 (2021).) co-existed in the N 1*s* spectrum.

The covalent Cl from C-Cl bond and ionic Cl (*Angew. Chem. Int. Ed.* **60**, 27324-27329 (2021).) co-existed in the Cl 2*p* spectrum.

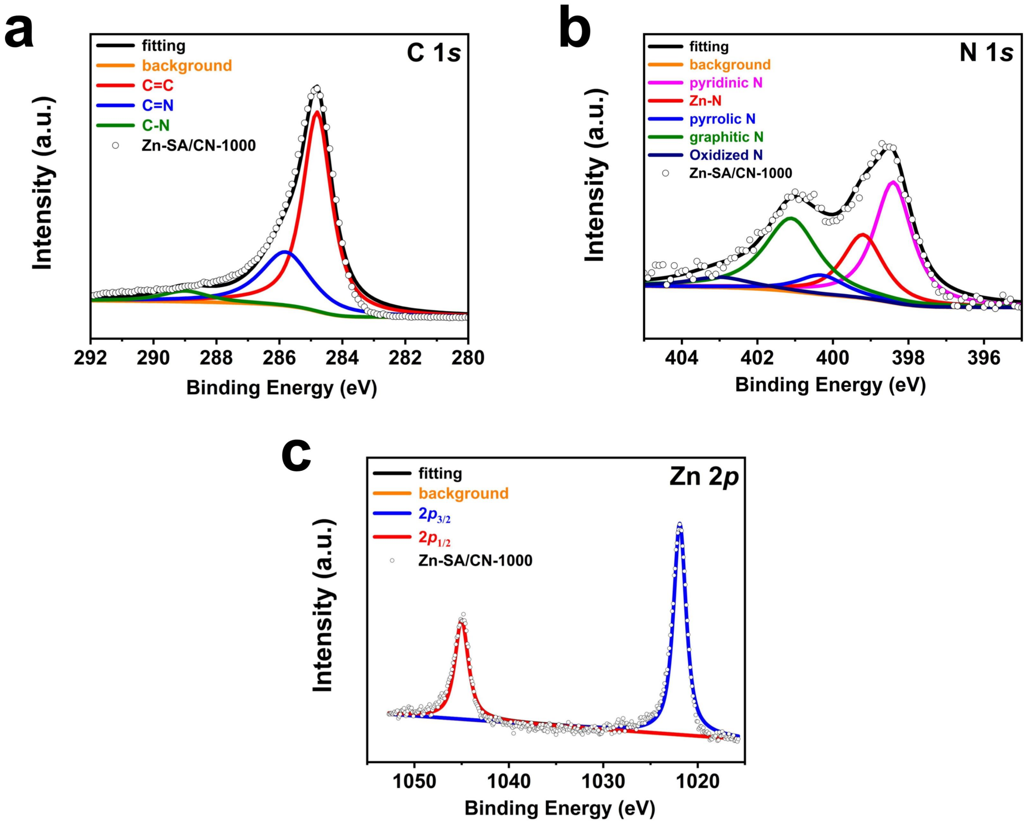

Supplementary Fig. 11. The XPS spectra of Zn-SA/CN-1000. **a**, XPS spectrum for the C 1*s*. **b**, XPS spectrum for the N 1*s*. **c**, XPS spectrum for the Zn 2*p*.

The C=C, C=N and C-N bonds (*Angew. Chem. Int. Ed.* **59**, 22465-22469 (2020).) co-existed in the C 1*s* spectrum.

The pyridinic N, Zn-N, pyrrolic N, graphitic N and oxidized N species (*Angew. Chem. Int. Ed.* **60**, 9078-9085 (2021).) co-existed in the N 1*s* spectrum.

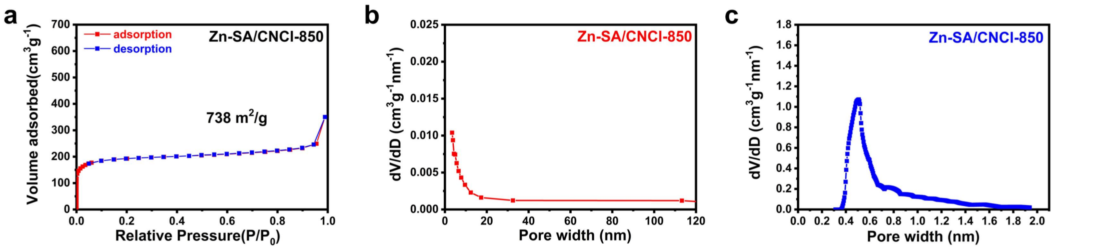

Supplementary Fig. 12. The BET surface area and pore size distribution of Zn-SA/CNCl-850. **a**, N_2 adsorption-desorption isotherms and corresponding BET surface area. **b**, The corresponding mesopore-size distribution. **c**, The corresponding micropore-size distribution.

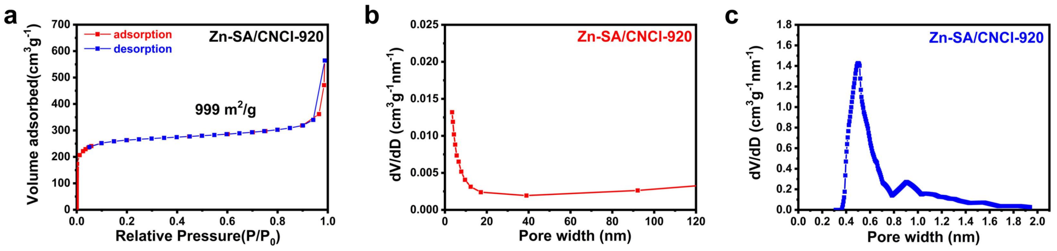

Supplementary Fig. 13. The BET surface area and pore size distribution of Zn-SA/CNCl-920. **a**, N_2 adsorption-desorption isotherms and corresponding BET surface area. **b**, The corresponding mesopore-size distribution. **c**, The corresponding micropore-size distribution.

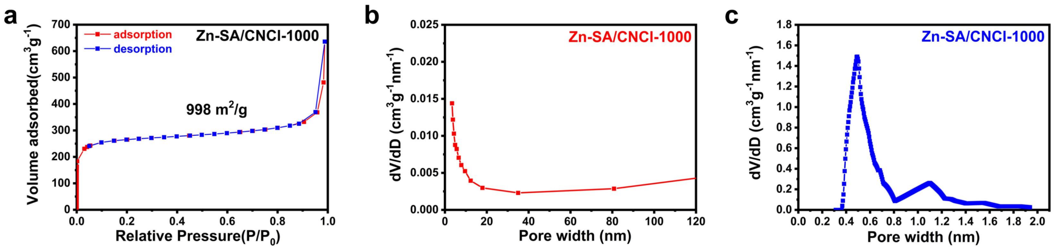

Supplementary Fig. 14. The BET surface area and pore size distribution of Zn-SA/CNCl-1000. **a**, N_2 adsorption-desorption isotherms and corresponding BET surface area. **b**, The corresponding mesopore-size distribution. **c**, The corresponding micropore-size distribution.

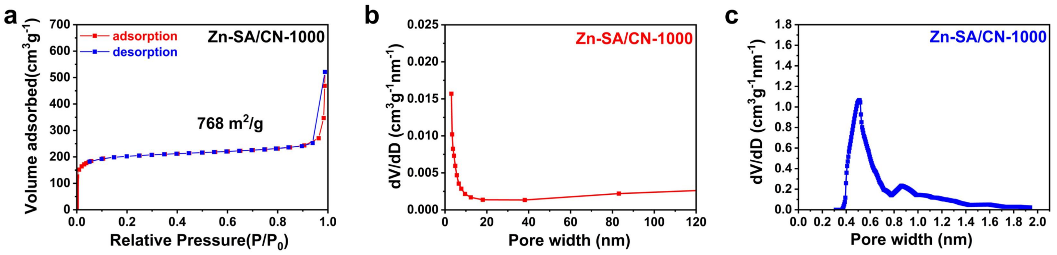

Supplementary Fig. 15. The BET surface area and pore size distribution of Zn-SA/CN-1000. **a**, N_2 adsorption-desorption isotherms and corresponding BET surface area. **b**, The corresponding mesopore-size distribution. **c**, The corresponding micropore-size distribution.

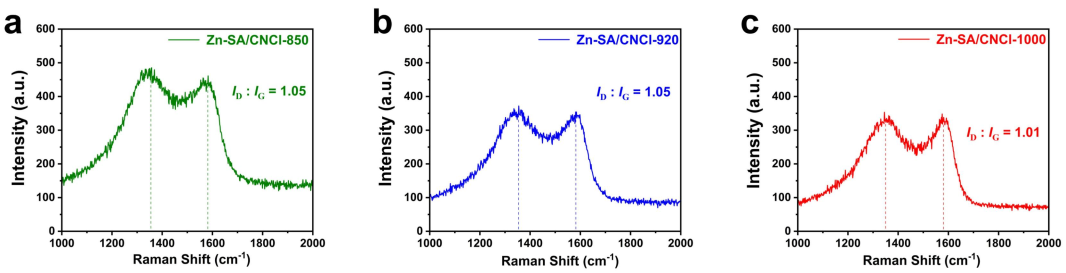

Supplementary Fig. 16. a-c, The Raman spectra of Zn-SA/CNCl-850, Zn-SA/CNCl-920 and Zn-SA/CNCl-1000, respectively.

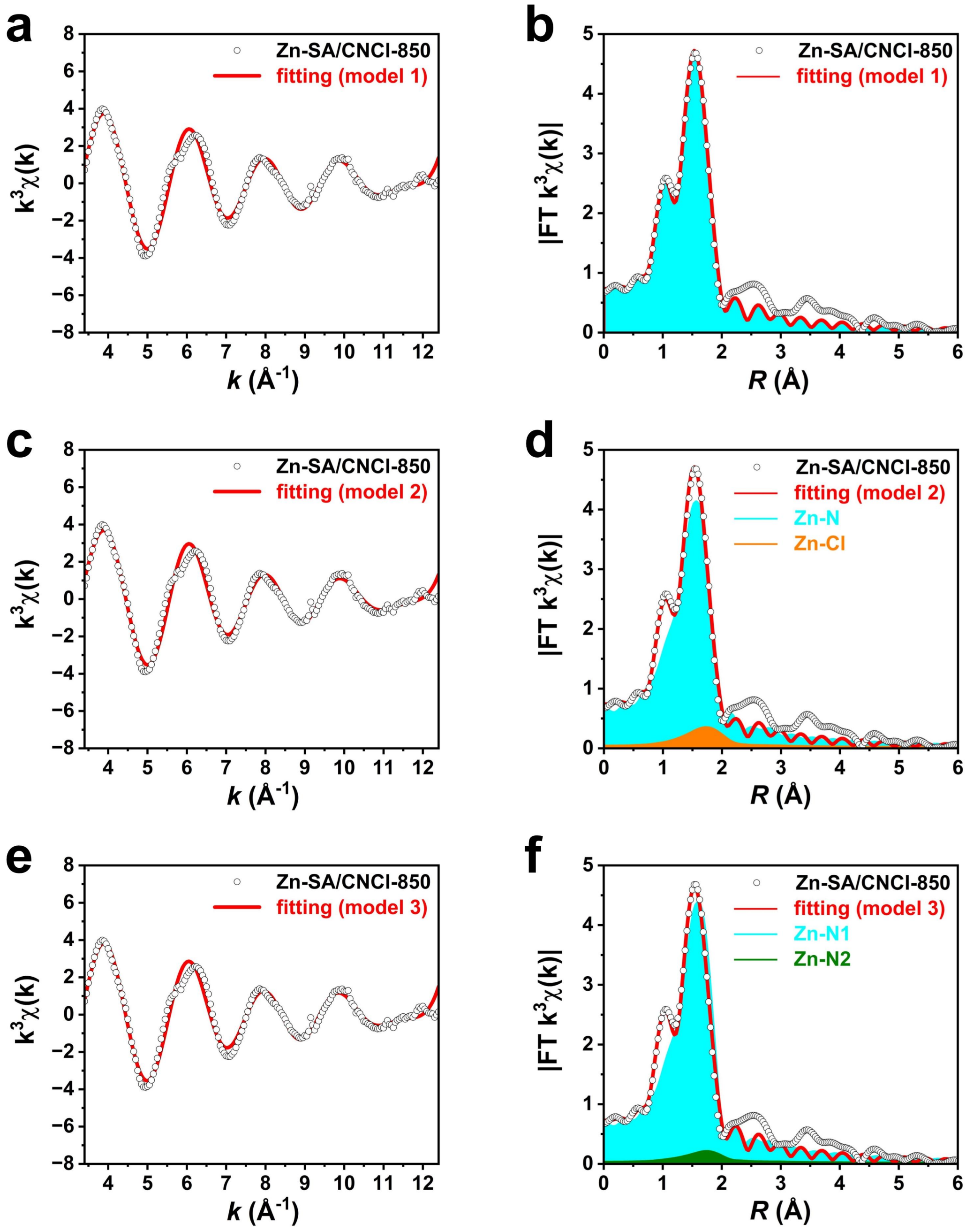

Supplementary Fig. 17. The EXAFS fitting results of Zn-SA/CNCl-850. **a-b**, The fitting result in *k* space and *R* space, respectively, with utilization of model 1 as fitting model. **c-d**, The fitting result in *k* space and *R* space, respectively, with utilization of model 2 as fitting model. **e-f**, The fitting result in *k* space and *R* space, respectively, with utilization of model 3 as fitting model. (**model 1**: with only one Zn-N path; **model 2**: with both Zn-N and Zn-Cl paths; **model 3**: with two non-equivalent Zn-N paths.)

The EXAFS fitting results of Zn-SA/CNCl-850 was shown in **Supplementary**

Fig. 17 and **Supplementary Table 2**. During fitting, the disorder factors of different paths were not constricted to the same value. As shown in **Supplementary Table 2**, the R factors of Zn-SA/CNCl-850 fitted by model 1, model 2 and model 3 are 0.001319, 0.001805 and 0.001919, respectively, indicating that the model 1 has the best goodness of fitting result for fitting Zn-SA/CNCl-850.

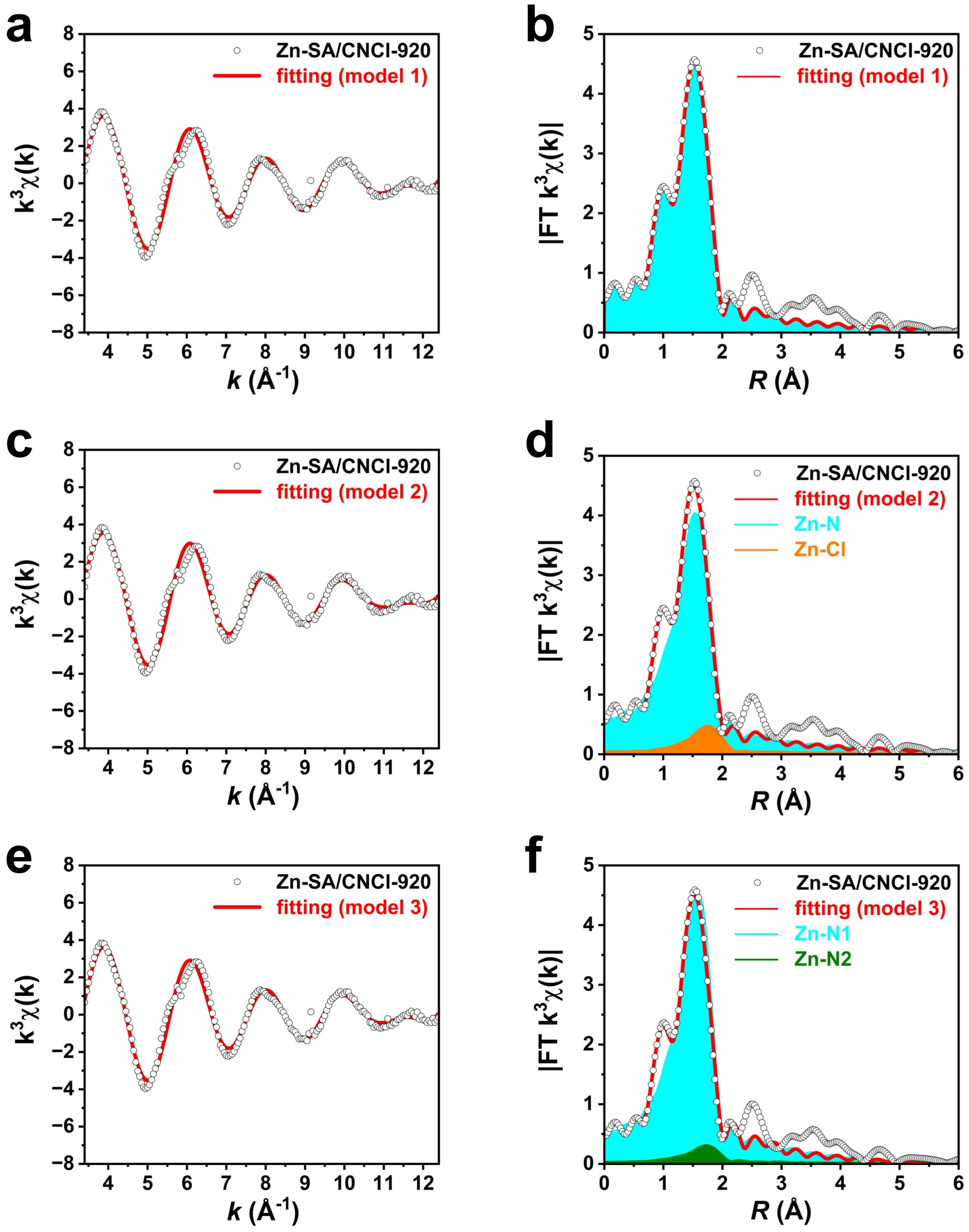

Supplementary Fig. 18. The EXAFS fitting results of Zn-SA/CNCl-920. **a-b**, The fitting result in *k* space and *R* space, respectively, with utilization of model 1 as fitting model. **c-d**, The fitting result in *k* space and *R* space, respectively, with utilization of model 2 as fitting model. **e-f**, The fitting result in *k* space and *R* space, respectively, with utilization of model 3 as fitting model. (**model 1**: with only one Zn-N path; **model 2**: with both Zn-N and Zn-Cl paths; **model 3**: with two non-equivalent Zn-N paths.)

The EXAFS fitting results of Zn-SA/CNCl-920 was shown in **Supplementary**

Fig. 18 and **Supplementary Table 3**. During fitting, the disorder factors of different paths were not constricted to the same value. As shown in **Supplementary Table 3**, the R factors of Zn-SA/CNCl-920 fitted by model 1, model 2 and model 3 are 0.000804, 0.001445 and 0.001520, respectively. Therefore, the model 1 is the best model for fitting Zn-SA/CNCl-920.

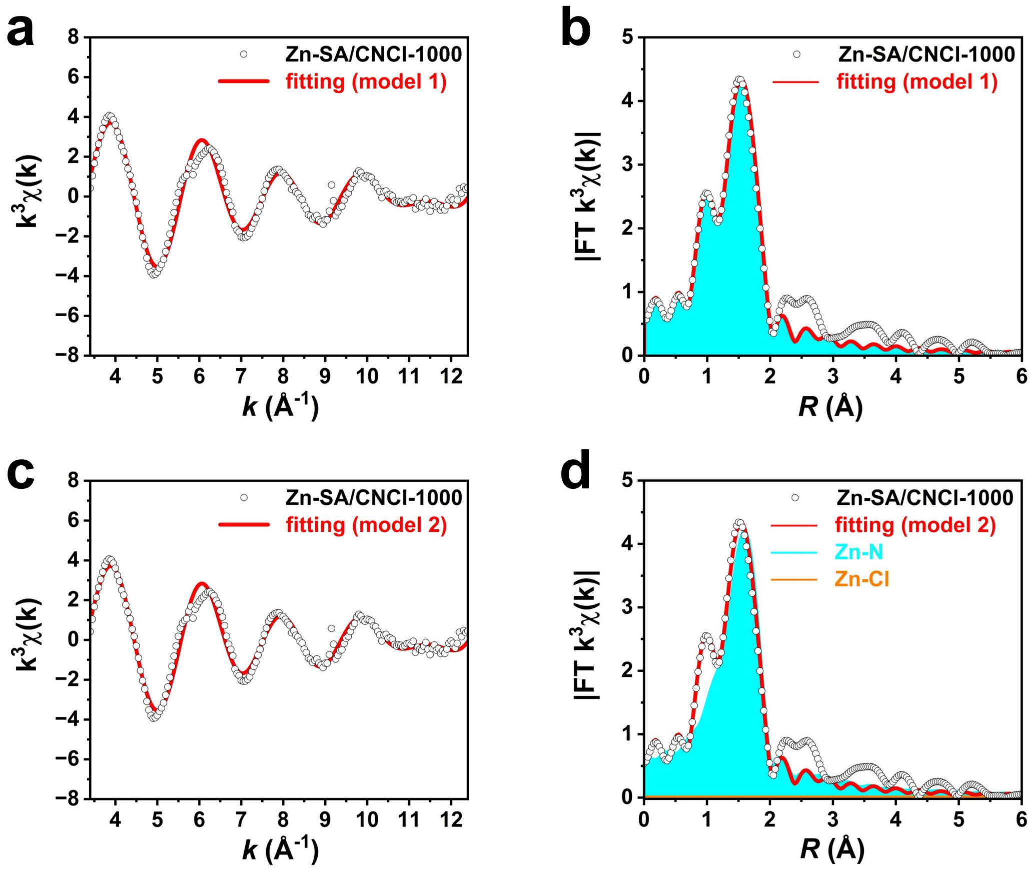

Supplementary Fig. 19. The EXAFS fitting results of Zn-SA/CNCl-1000. **a-b**, The fitting result in *k* space and *R* space, respectively, with utilization of model 1 as fitting model. **c-d**, The fitting result in *k* space and *R* space, respectively, with utilization of model 2 as fitting model. (**model 1**: with only one Zn-N path; **model 2**: with both Zn-N and Zn-Cl paths.)

The EXAFS fitting results of Zn-SA/CNCl-1000 was shown in **Supplementary Fig. 19** and **Supplementary Table 4**. During fitting, the disorder factors of different paths were not constricted to the same value. As shown in **Supplementary Table 4**, the R factors of Zn-SA/CNCl-1000 fitted by model 1 and model 2 are 0.002235 and 0.002278, respectively, which are similar to each other. After adding the Zn-Cl path for fitting Zn-SA/CNCl-1000 by model 2, the coordination number of Zn-Cl bond in model 2 is 0.00 ± 0.17, indicating the Zn-Cl bond is negligible in the Zn-SA/CNCl-1000. Therefore, the model 1 with only Zn-N path is the rational model for fitting Zn-SA/CNCl-1000.

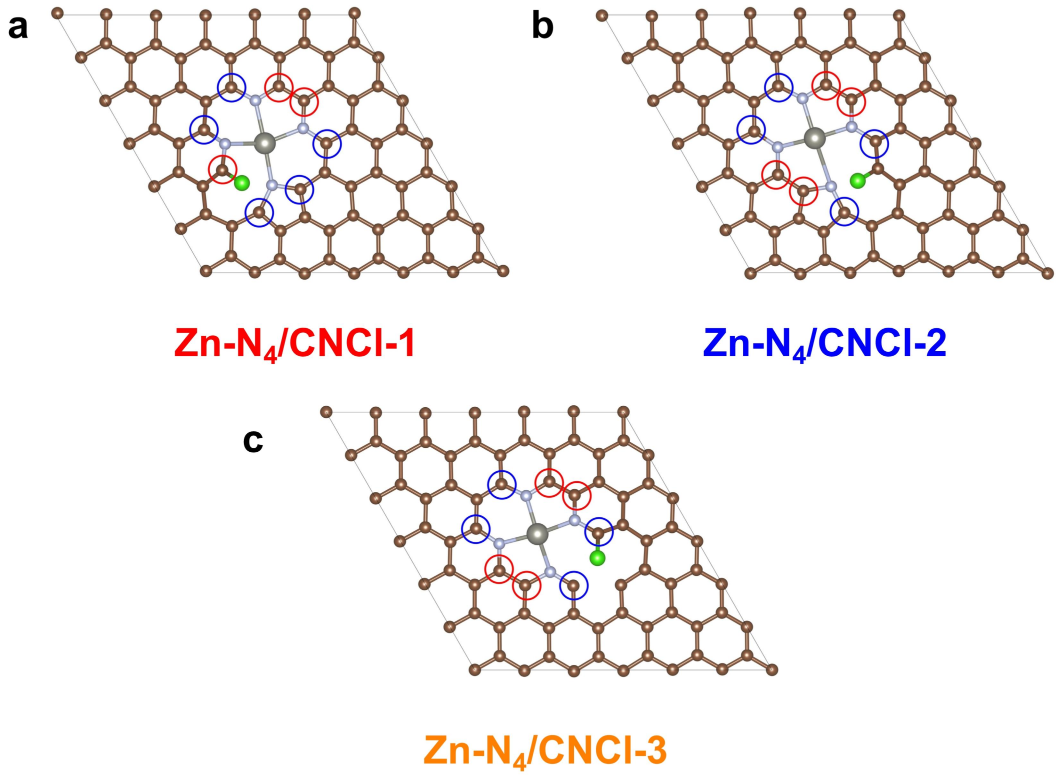

Supplementary Fig. 20. a-c, The analysis the coordinate structure of Zn atom in Zn-N_4/CNCl-1, Zn-N_4/CNCl-2 and Zn-N_4/CNCl-3 models, respectively.

As shown in **Supplementary Fig. 20**, we analyzed the coordinate structure of Zn atom of the DFT models in **Figure 5a**. As shown in **Supplementary Fig. 20a**, four N atoms located in the first coordination shell of Zn atom in Zn-N_4/CNCl-1 model. While eight C atoms located in the second coordination shell of Zn atom. There were two non-equivalent Zn-C paths with three shorter Zn-C distance marked in red circles and five longer Zn-C distance marked in blue circles. Theoretically, the Cl atom located in the third coordination shell of Zn atom with Zn-N-C-Cl structure.

As shown in **Supplementary Fig. 20b**, four N atoms located in the first coordination shell of Zn atom in Zn-N_4/CNCl-2 model. While eight C atoms located in the second coordination shell of Zn atom. There were two non-equivalent Zn-C paths with four shorter Zn-C distance marked in red circles and four longer Zn-C distance marked in blue circles. Theoretically, the Cl atom located in the fourth coordination shell of Zn atom with Zn-N-C-C-Cl structure.

As shown in **Supplementary Fig. 20c**, four N atoms located in the first

coordination shell of Zn atom in Zn-N_4/CNCl-3 model. While eight C atoms located in the second coordination shell of Zn atom. There were two non-equivalent Zn-C paths with four shorter Zn-C distance marked in red circles and four longer Zn-C distance marked in blue circles. Theoretically, the Cl atom located in the third coordination shell of Zn atom with Zn-N-C-Cl structure.

Therefore, theoretically, only two non-equivalent Zn-C paths should be considered for fitting the second coordination shell of Zn atom in Zn-N_4/CNCl models without Zn-Cl path. However, considering the Zn-Cl distance was comparable to that of Zn-C paths, fitting the second coordination shell of Zn atom in Zn-N_4/CNCl models with both Zn-C path and Zn-Cl path was also achievable.

When considering both the Zn-C path and Zn-Cl path as the second coordination shell of Zn atom, the main contribution of the second coordination shell of Zn atom was from Zn-C path rather than Zn-Cl path since eight C atoms and only one Cl atom were in the second coordination shell of Zn atom. Therefore, simultaneously fitting the Zn-C path and Zn-Cl path in the second coordination shell of Zn atom is challenging because the contribution of Zn-C path is much stronger that of Zn-Cl path. Thus, on the background of strong contribution of Zn-C path, obtaining the accurate coordination number of Zn-Cl path is challenging because the contribution of Zn-C path can easily cover the contribution of Zn-Cl path.

Besides, the Zn-SA/CNCl catalysts lacked the periodic crystal structure like inorganic crystal and the chemical structure of the second coordination shell of Zn atom was irregular. The Cl atoms were randomly located in the second or higher coordination shells of Zn atom. Therefore, simultaneously fitting Zn-C and Zn-Cl paths as the second coordination shell of Zn atom and obtaining the accurate coordination information of Zn-Cl path, such as coordination number and Zn-Cl distance is challenging with larger errors.

Thus, only two non-equivalent Zn-C paths were considered as the second coordination shell of Zn atom. The fitting results were exhibited in **Supplementary Fig. 21** and **Supplementary Table 5**.

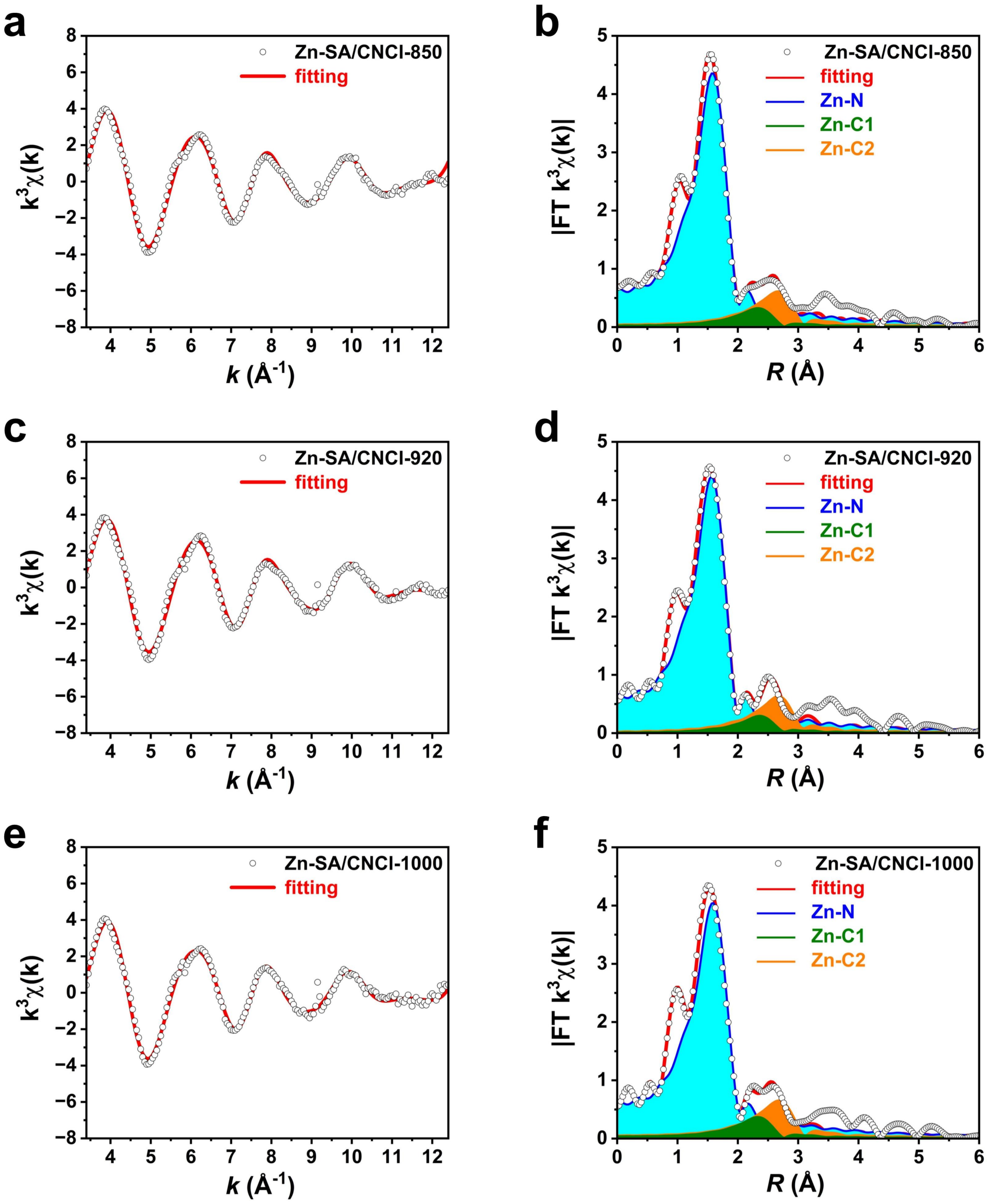

Supplementary Fig. 21. The FT-EXAFS fitting results of the first and second coordination shells of Zn atom in Zn-SA/CNCl catalysts. **a-b**, The fitting result of Zn-SA/CNCl-850 in *k* space and *R* space, respectively. **c-d**, The fitting result of Zn-SA/CNCl-920 in *k* space and *R* space, respectively. **e-f**, The fitting result of Zn-SA/CNCl-1000 in *k* space and *R* space, respectively.

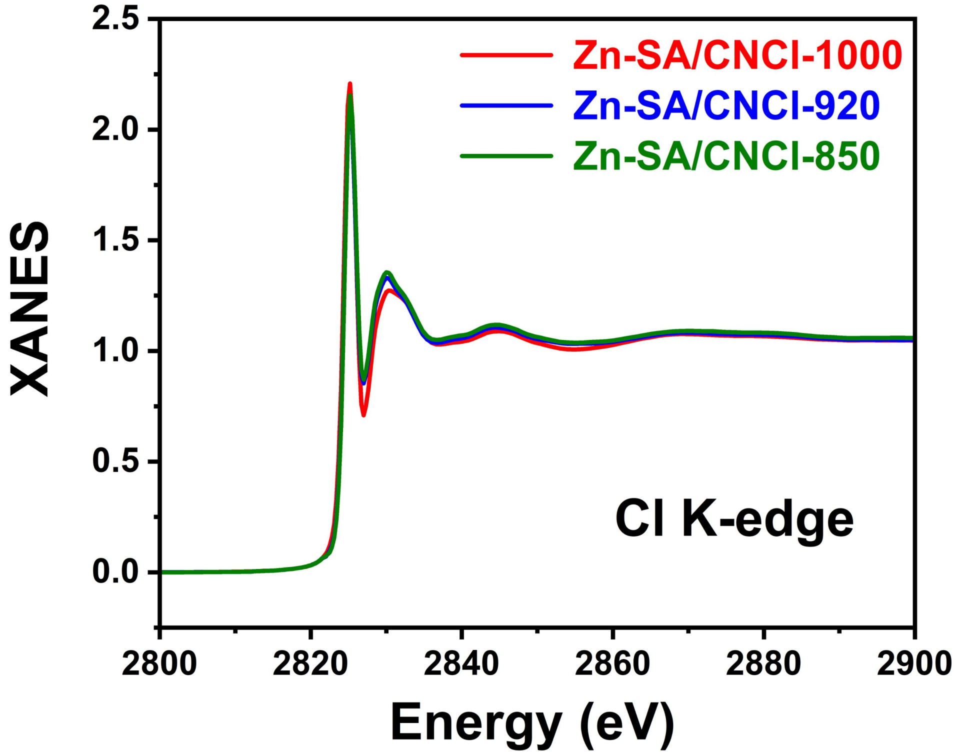

Supplementary Fig. 22. The XANES spectra of Zn-SA/CNCl catalysts at Cl K-edge.

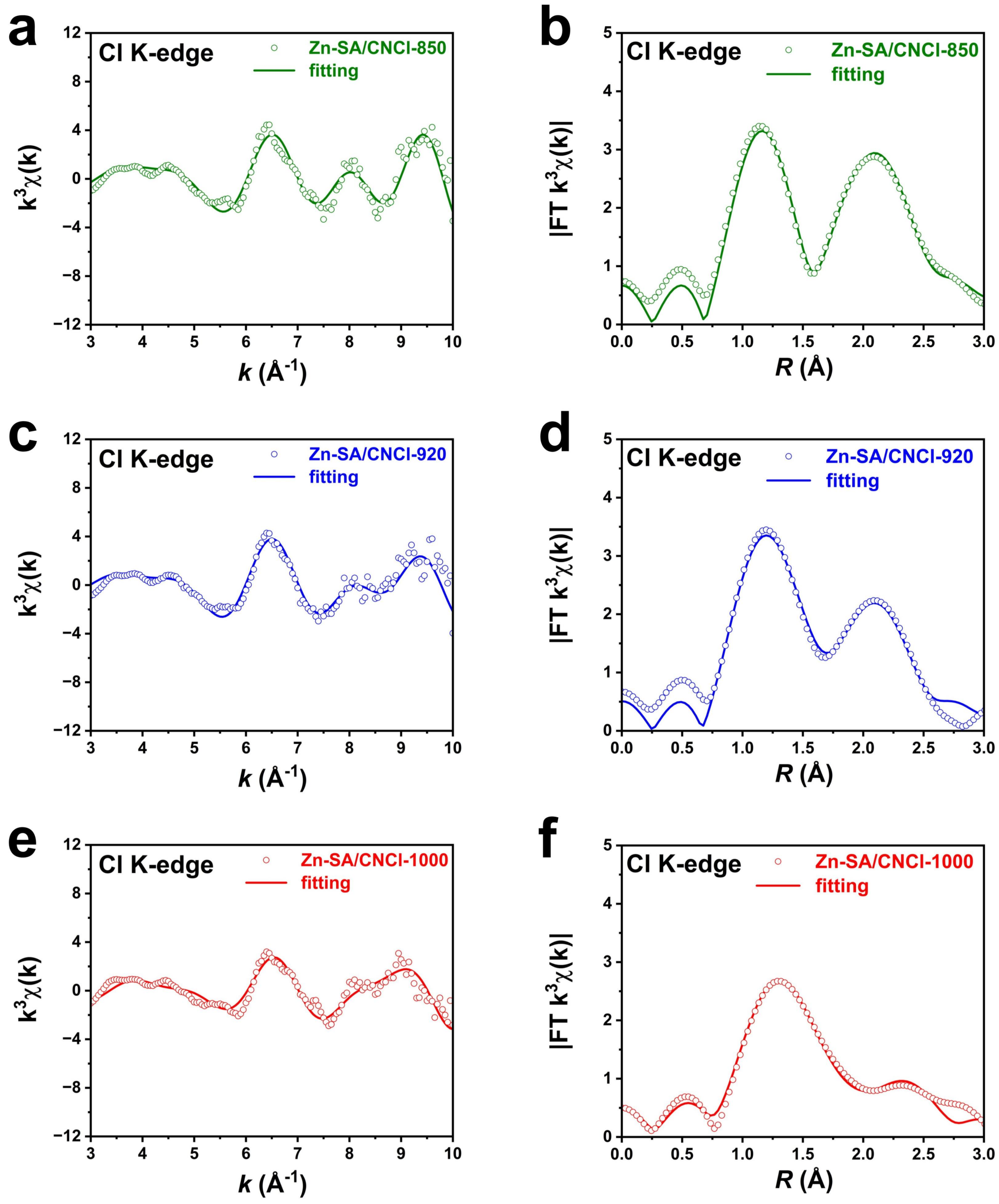

Supplementary Fig. 23. a-b, The fitting results of Zn-SA/CNCl-850 at Cl K-edge in *R* space and *k* space, respectively. **c-d**, The fitting results of Zn-SA/CNCl-920 at Cl K-edge in *R* space and *k* space, respectively. **e-f**, The fitting results of Zn-SA/CNCl-1000 at Cl K-edge in *R* space and *k* space, respectively.

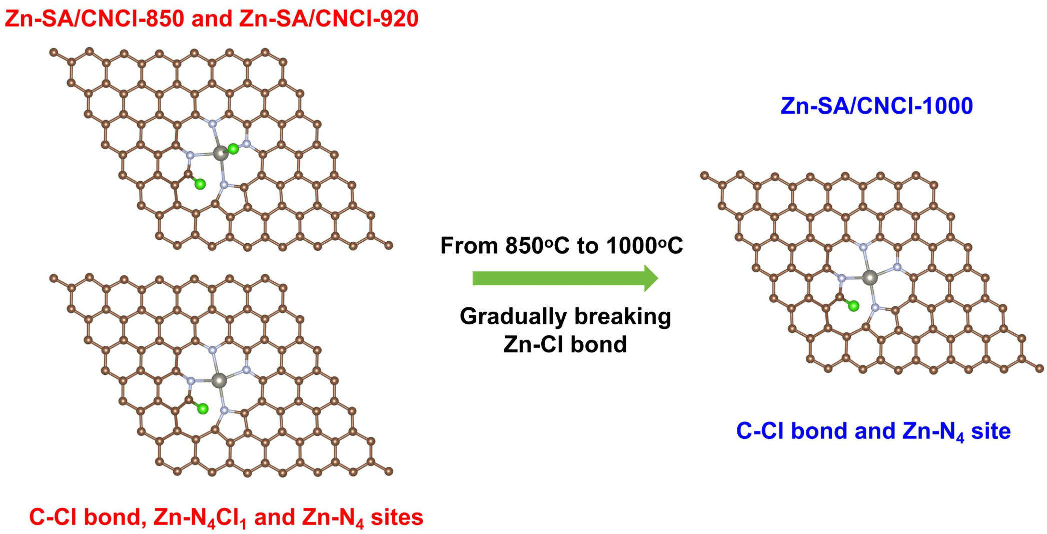

Supplementary Fig. 24. The evolution of catalytic site from Zn-SA/CNCl-850 to Zn-SA/CNCl-1000 catalysts.

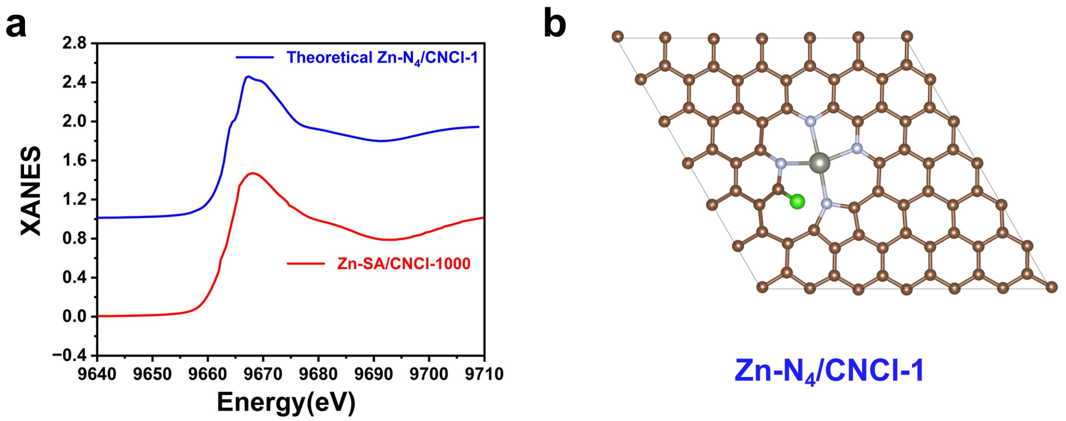

Supplementary Fig. 25. a, The XANES spectrum of Zn-SA/CNCl-1000 and the theoretical spectrum of Zn-N_4/CNCl-1 model. **b**, The corresponding Zn-N_4/CNCl-1 model.

We also simulate the XANES curve of the optimized model of Zn-SA/CNCl-1000 in **Supplementary Fig. 24**. As shown in **Supplementary Fig. 25a**, the XANES spectrum of Zn-SA/CNCl-1000 and the theoretical spectrum of Zn-N_4/CNCl-1 model (**Supplementary Fig. 25b**) exhibited similar features, revealing the Zn-N_4/CNCl-1 model was a rational model to simulate the Zn-SA/CNCl-1000.

We utilized FDMNES with the 29th reversion to simulate the XANES spectra. We calculated the theoretical XANES spectra by using all the software's default options within the finite difference method mode. Upon entering the lattice parameters and atomic structure into the software, we incrementally calculated the spectrum with varying "Cluster Radius" from 3 to 7 angstrom and convoluted the spectra.

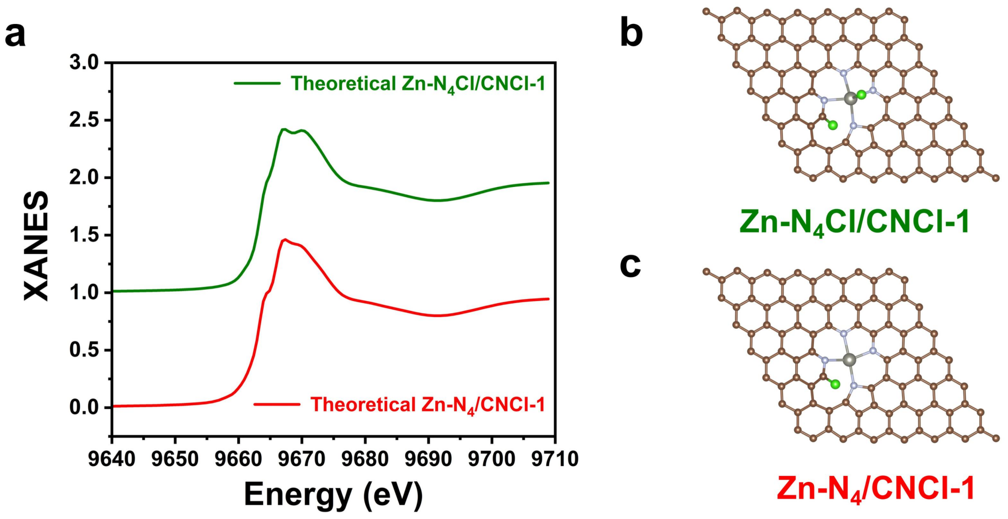

Supplementary Fig. 26. a, The theoretical XANES spectra of Zn-N_4Cl/CNCl-1 and Zn-N_4/CNCl-1 models. **b-c**, The corresponding optimized Zn-N_4Cl/CNCl-1 and Zn-N_4/CNCl-1 models.

As shown in **Supplementary Fig. 26a**, the theoretical XANES spectrum of Zn-N_4Cl/CNCl-1 model with Zn-N_4Cl_1 site is similar to that of Zn-N_4/CNCl-1 model with Zn-N_4 site. Therefore, the gradual transformation from the Zn-N_4Cl_1 site into the Zn-N_4 site can not induce remarkable change of XANES spectrum at Zn-K edge.

Besides, the Cl element is in trace amount in Zn-SA/CNCl catalysts and the Zn element in Zn-SA/CNCl catalysts mainly exists as Zn-N bonds. Therefore, the effect of trace amount Cl element on the XANES spectra of Zn element in Zn-SA/CNCl catalysts is not remarkable. Therefore, the experimental XANES spectra of Zn-SA/CNCl-850, Zn-SA/CNCl-920 and Zn-SA/CNCl-1000 are similar to each other.

We utilized FDMNES with the 29th reversion to simulate the XANES spectra. We calculated the theoretical XANES spectra by using all the software's default options within the finite difference method mode. Upon entering the lattice parameters and atomic structure into the software, we incrementally calculated the spectrum with varying "Cluster Radius" from 3 to 7 angstrom and convoluted the spectra.

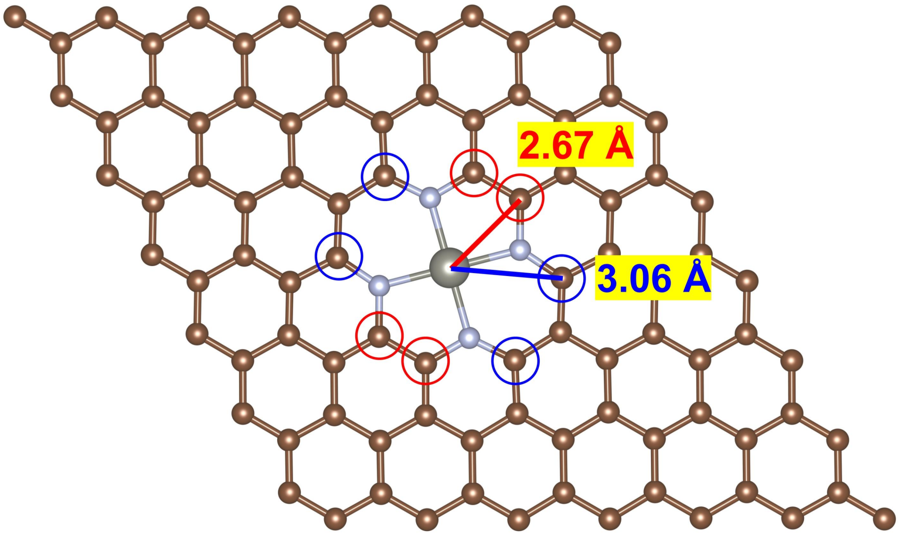

Supplementary Fig. 27. The optimized Zn-N_4/CN model to simulate the Zn-SA/CN-1000 without Cl-doping.

As shown in **Supplementary Fig. 27**, we utilized the Zn-N_4/CN model to simulate the Zn-SA/CN-1000 without Cl-doping. The first coordination shell of Zn atom was four N atoms. The second coordination shell of Zn atom included eight C atoms, with four C atoms with shorter Zn-C distance (2.67 Å) marked by red circles and four C atoms with longer Zn-C distance (3.06 Å) marked by blue circles.

Therefore, we utilized two non-equivalent Zn-C paths to fit the second coordination shell of Zn atom in Zn-SA/CN-1000 without Cl-doping. The fitting results were shown in **Supplementary Fig. 28** and **Supplementary Table 7**.

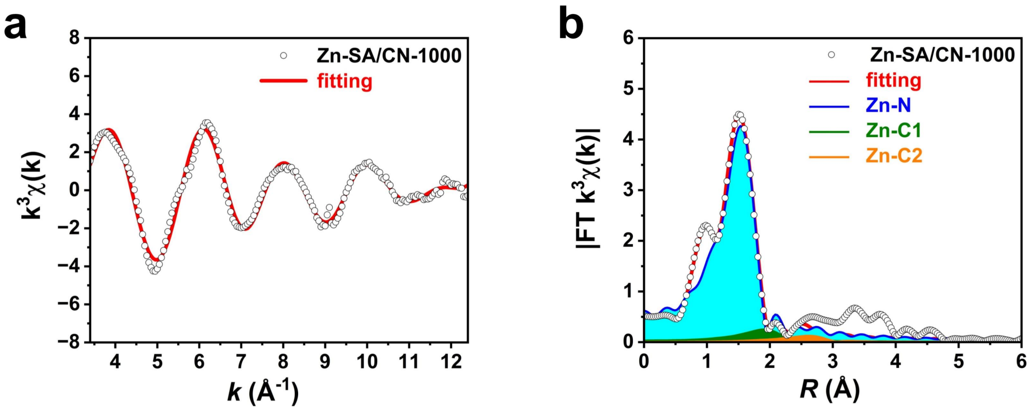

Supplementary Fig. 28. The FT-EXAFS fitting results of the first and second coordination shells of Zn atom in Zn-SA/CN-1000. **a**, The fitting result in *k* space. **b**, The fitting result in *R* space.

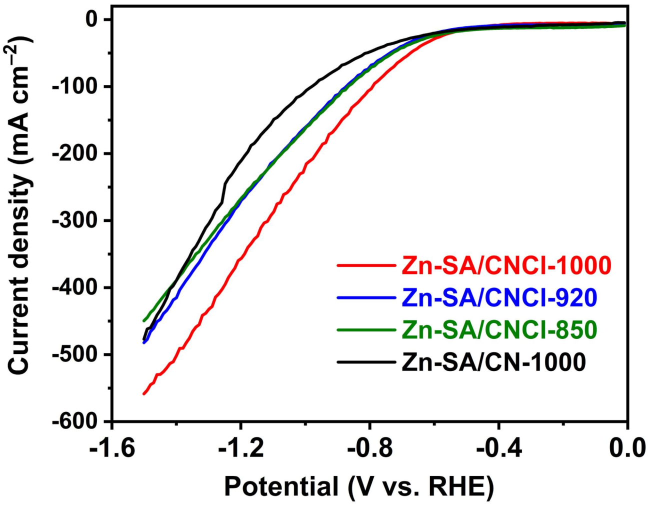

Supplementary Fig. 29. The comparison of total current density for CO_2RR catalyzed by Zn-based catalysts without iR correction.

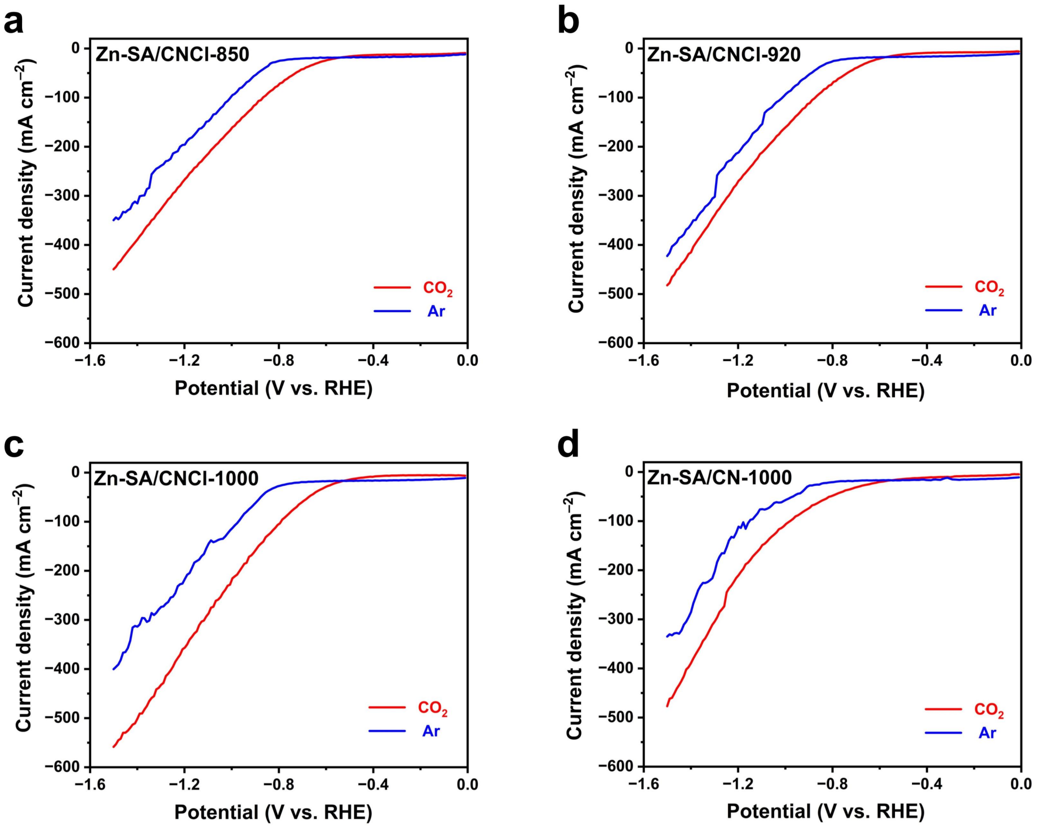

Supplementary Fig. 30. a-d, The comparison of LSV curves without iR correction in flow cell electrolyzer with flowing Ar or CO_2 catalyzed by Zn-SA/CNCl-850, Zn-SA/CNCl-920, Zn-SA/CNCl-1000 and Zn-SA/CN-1000, respectively.

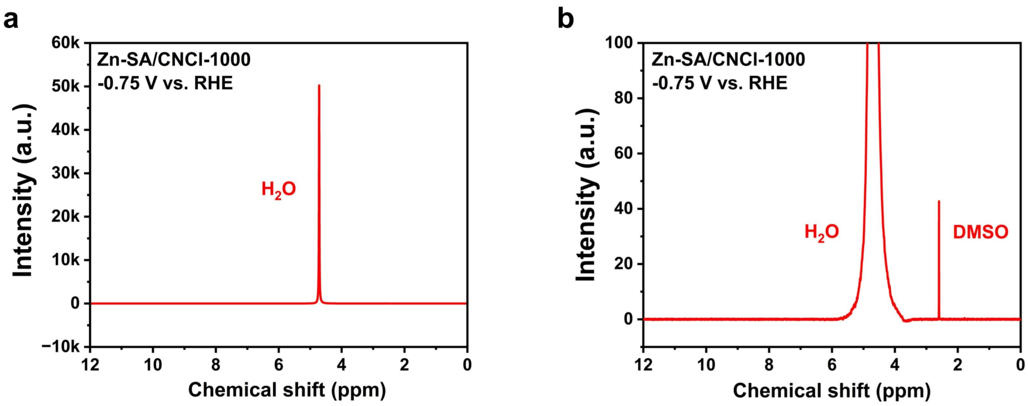

Supplementary Fig. 31. a, The NMR spectrum to detect liquid products catalyzed by Zn-SA/CNCl-1000 catalyst at -0.75 V vs. RHE. (The potential was provided after iR correction.) **b**, The enlarged NMR spectrum. Only the signals of H_2O (solvent) and DMSO (reference) appeared and no liquid products was found.

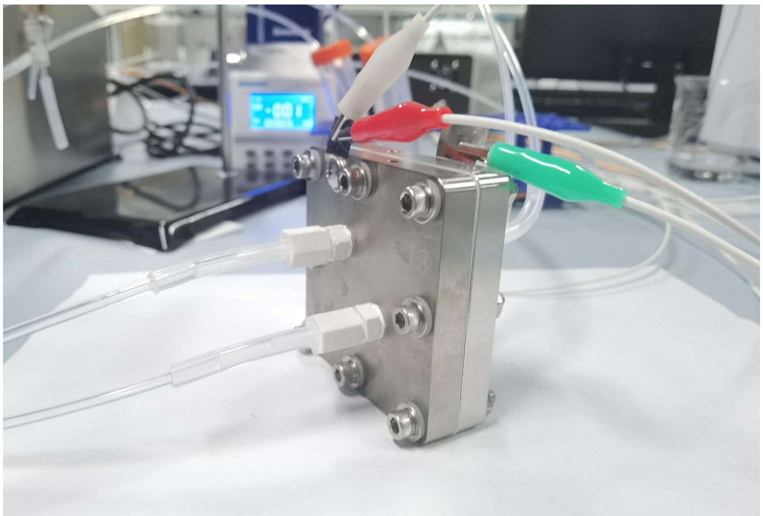

Supplementary Fig. 32. The photograph of membrane electrode assembly (MEA).

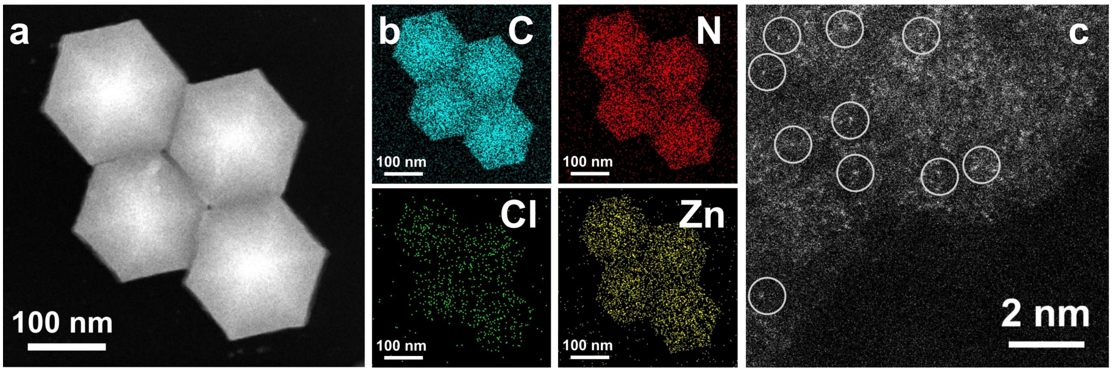

Supplementary Fig. 33. a-b, The HAADF-STEM image of Zn-SA/CNCl-1000 after CO_2RR and the corresponding EDX spectroscopy elemental mapping results. **c**, The AC-STEM image of Zn-SA/CNCl-1000 after CO_2RR.

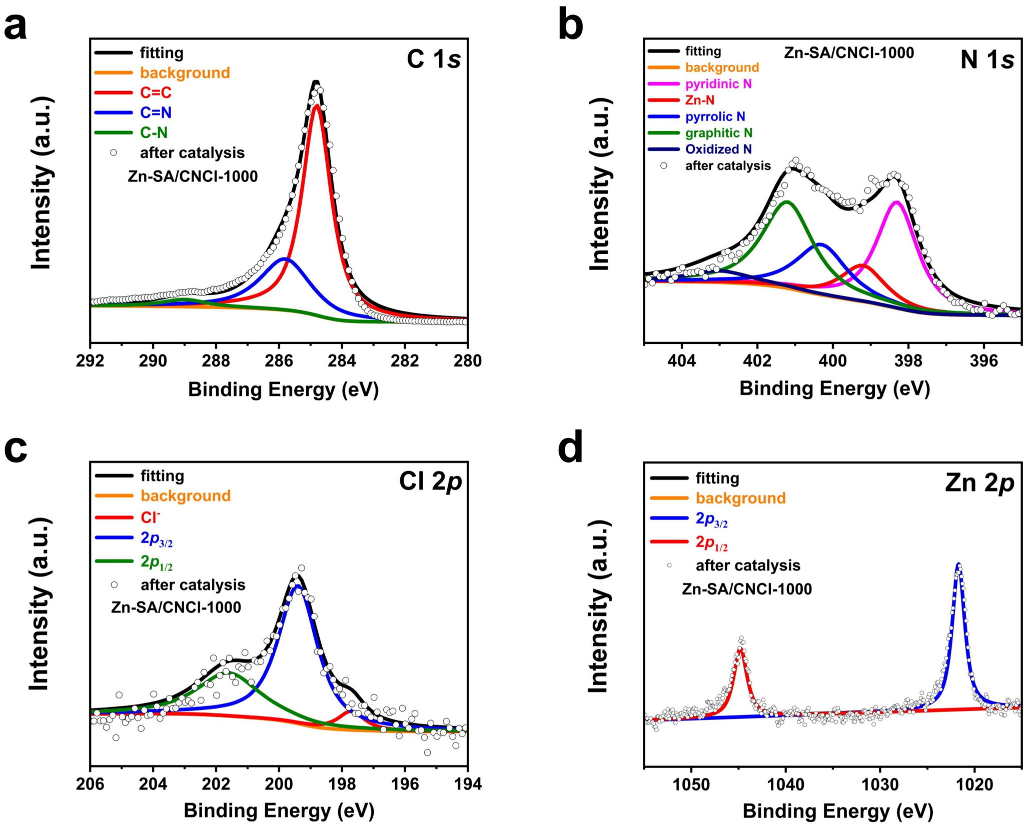

Supplementary Fig. 34. The XPS spectra of Zn-SA/CNCl-1000 after CO_2RR. **a**, XPS spectrum for the C 1*s*. **b**, XPS spectrum for the N 1*s*. **c**, XPS spectrum for the Cl 2*p*. **d**, XPS spectrum for the Zn 2*p*.

The C=C, C=N and C-N bonds (*Angew. Chem. Int. Ed.* **59**, 22465-22469 (2020).) co-existed in the C 1*s* spectrum.

The pyridinic N, Zn-N, pyrrolic N, graphitic N and oxidized N species (*Angew. Chem. Int. Ed.* **60**, 9078-9085 (2021).) co-existed in the N 1*s* spectrum.

The covalent Cl from C-Cl bond and ionic Cl (*Angew. Chem. Int. Ed.* **60**, 27324-27329 (2021).) co-existed in the Cl 2*p* spectrum.

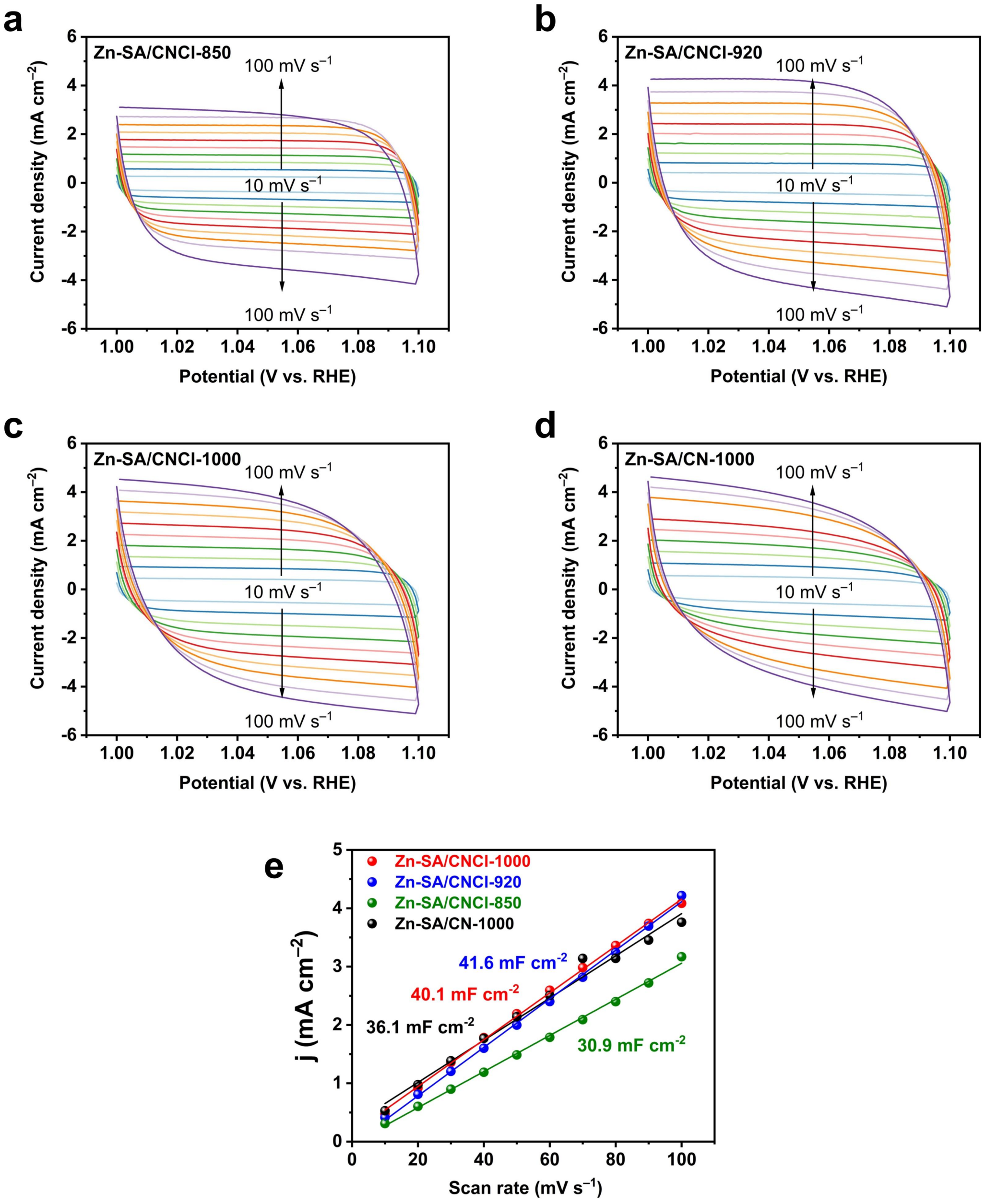

Supplementary Fig. 35. a-d, The CV curves with different scan rates under the potential of 1.00 V- 1.10 V vs. RHE without iR correction of Zn-SA/CNCl-850, Zn-SA/CNCl-920, Zn-SA/CNCl-1000 and Zn-SA/CN-1000, respectively. **e**, Δj at 1.05 V vs. RHE as a function of the scan rates to evaluate the double-layer capacitor (C_{dl}). The C_{dl} of Zn-SA/CNCl-850, Zn-SA/CNCl-920, Zn-SA/CNCl-1000 and Zn-SA/CN-1000 were 30.9 mF cm^{-2}, 41.6 mF cm^{-2}, 40.1 mF cm^{-2}, and 36.1 mF cm^{-2}, respectively. These results indicated that the Zn-SA/CNCl-1000, Zn-SA/CNCl-920 and Zn-SA/CN-1000 had similar ECSAs, higher than that of Zn-SA/CNCl-850.

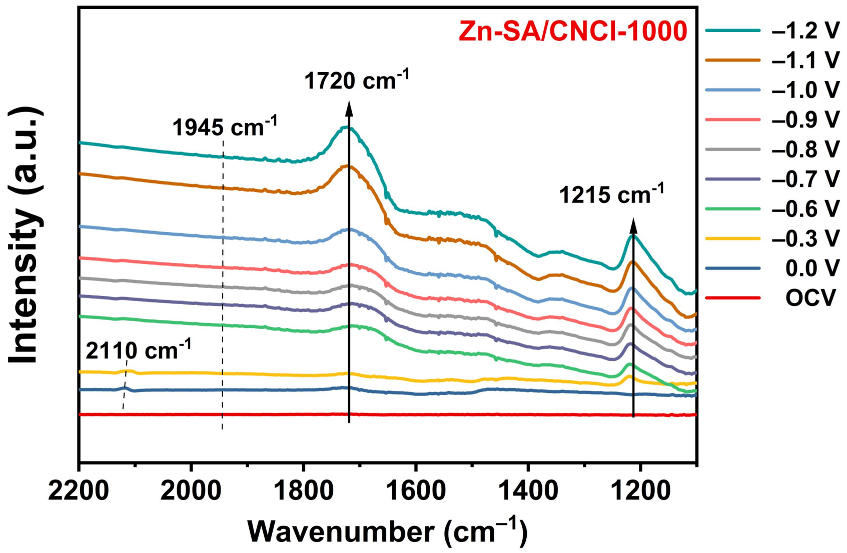

Supplementary Fig. 36. The in situ ATR-SEIRAS measurement for CO_2RR catalyzed by Zn-SA/CNCl-1000 at OCV, 0.0 V, -0.3 V, -0.6 V, -0.7 V, -0.8 V, -0.9 V, -1.0 V, -1.1 V, and -1.2 V vs. RHE. The potentials are provided without iR correction.

When Si prism is used as the IR window, the oxide layer on the surface of Si prism also has adsorption peaks, such as the adsorption peaks of Si-O-Si and Si-OH. As reported by the reference (*Journal of Cleaner Production* **2022**, *380*, 134975), the band of the vibration of Si-O-Si bonds located at 1095 cm^{-1} while the band of the stretching vibration of Si-OH appeared at 950 cm^{-1}. As reported by the reference (*Appl. Phys. A* **2018**, *124*, 802), the IR characteristic peaks of the Si-O-Si bond are all strong stretching vibration absorption peaks, which appear at 527 cm^{-1}, 465 cm^{-1}, 1095 cm^{-1} and 1020 cm^{-1}. Thus, we analyze the peaks distribution above 1100 cm^{-1} to avoid the disruptions of Si signals. Besides, in the pre-treatment of Si prism, we have used 40% NH_4F to remove the oxide layer on Si surface.

As shown in **Supplementary Fig. 36**, we further analyze the adsorption range to 2200 cm^{-1}. At the potential of 0 V and -0.3 V vs. RHE, a adsorption at 2110 cm^{-1} was observed, which is attributed the terminally CO (CO_{atop}). This peak quickly disappeared when the applied potential shift to -0.6 V vs. RHE because the CO_{atop} was quickly desorbed from electrode surface and was converted into gaseous CO, which

was favourable for the formation of gaseous CO as main product.

Additionally, from -0.6 V to -1.2 V vs. RHE, the peak intensities at 1945 cm^{-1} corresponding to bridge CO (CO_{bridge}) were rather weak, without obvious enhancement as the potential decreased from -0.6 V to -1.2 V vs. RHE. As confirmed by references (*Proc. Natl. Acad. Sci. USA.* **2016**, *113*, E4585-E4593; *Nano Lett.* **2019**, *19*, 4817-4826), the CO_{bridge} is inert intermediate and hard to be converted to gaseous CO, while CO_{atop} is active and can be quickly desorbed. Therefore, we conclude that CO_2RR on Zn-SA/CNCl-1000 proceeds through CO_{atop} rather than CO_{bridge}, facilitating the formation of gaseous CO as main product, which was consistent with the experimental results for CO_2RR.

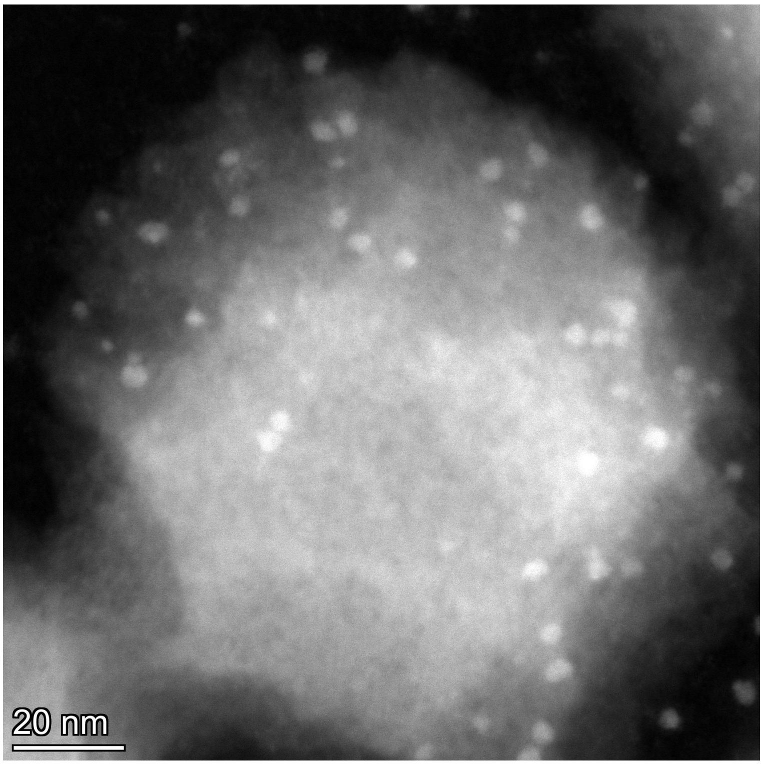

Supplementary Fig. 37. The HAADF-STEM image of ZnO_x/CN catalyst.

The ZnO_x/CN catalyst was synthesized by heating the Zn-SA/CN-1000 catalyst at 350°C for 1h in air with heating rate of 5°C/min. As shown in **Supplementary Fig. 37**, ZnO_x clusters appeared on the N-doped carbon substrate after heating Zn-SA/CN-1000 catalyst in air.

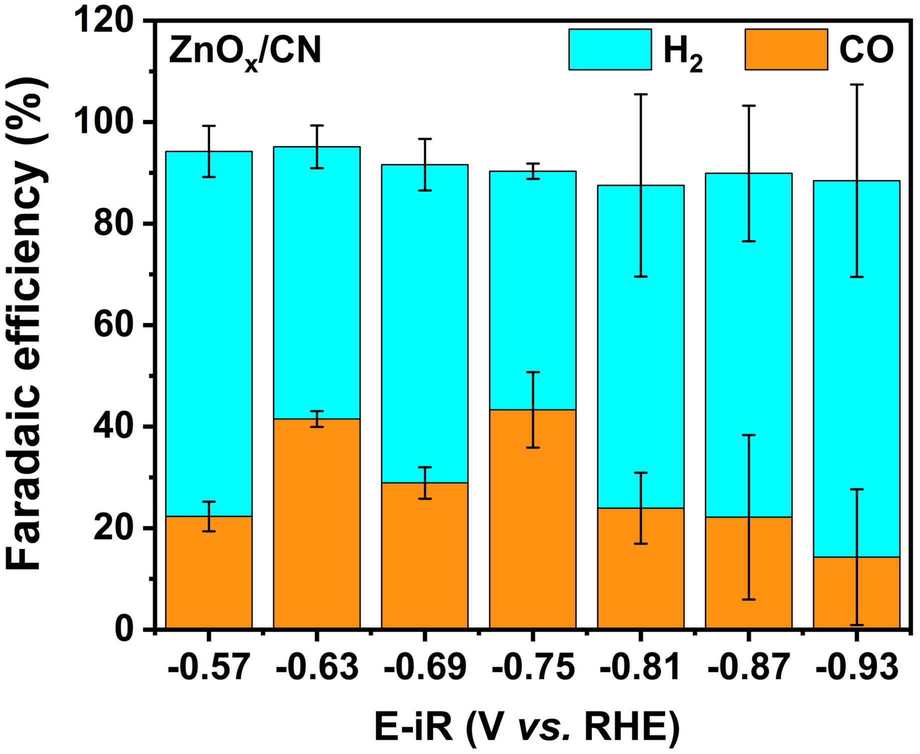

Supplementary Fig. 38. The faradaic efficiency of CO for CO_2RR catalyzed by ZnO_x/CN at different potentials. The potentials are provided after iR correction.

We also tested the catalytic performance of ZnO_x/CN catalyst for CO_2RR. As shown in **Supplementary Fig. 38**, the main product for CO_2RR catalyzed by ZnO_x/CN was H_2 rather than CO, ranging from -0.57 V to -0.93 V *vs.* RHE. The maximum faradaic efficiency of CO catalyzed by ZnO_x/CN was 43.31%, achieved at -0.75 V *vs.* RHE. These results revealed the poor catalytic activity of ZnO_x/CN catalyst for CO_2RR. The Error bars represent s.d. obtained from three independent experiments.

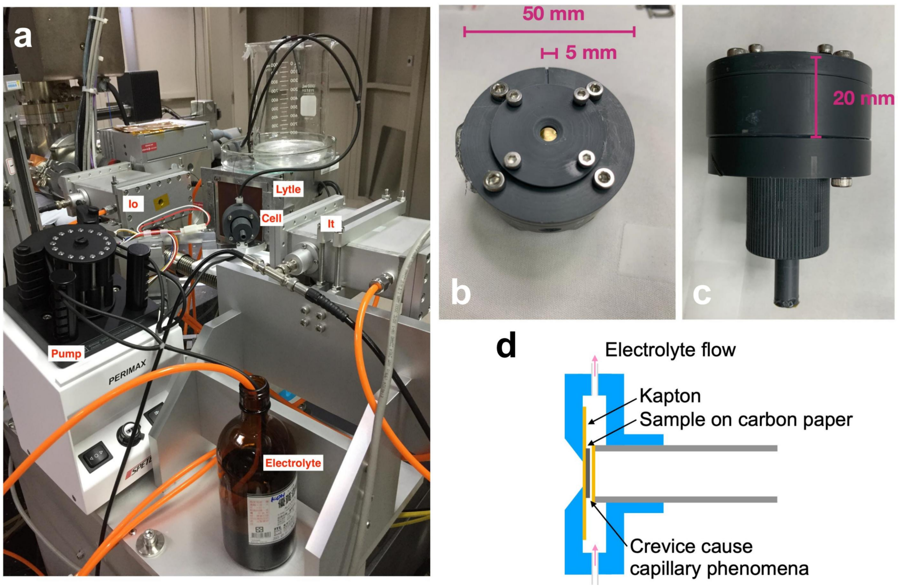

Supplementary Fig. 39. a, The photograph of the in-situ EXAFS experiments for CO_2RR. **b-c**, The photographs of the in-situ cell. **d**, The schematic diagram of capillary action, allowing for quick removal of electrochemically generated bubbles.

As shown in **Supplementary Fig. 39a**, to conduct our in-situ EXAFS experiments for CO_2RR, we set up three ionization chambers for I_0, I_t, and I_f, respectively, similar to the regular setup. A Lytle detector was employed to measure the fluorescence signals. The Lytle detector is also designed as an ionization chamber with a large collection area. It contains a fan-shaped slit to block the scattered X-ray and a filter to reduce the K_β fluorescence signals (In our Zn K-edge case, Cu foil was used for the filter). The in-situ cell was mounted between the I_0 and I_t chambers and tilted 45 degrees toward the fluorescence detector. Outside the cell, CO_2 gas was introduced into the 0.5 M $KHCO_3$ electrolyte at a rate of 60 c.c./min for 30 minutes to produce a saturated solution and kept the flow throughout the entire experiment.

As shown in **Supplementary Fig. 39d**, the peristaltic pump continuously delivers CO_2-saturated electrolytes into the cell from the bottom side and exits from above. We coated the sample on carbon paper and covered it with a Kapton window. An X-ray penetrable tube is inserted close to the sample, leaving a small gap. This small gap will create capillary action, allowing for quick removal of electrochemically generated bubbles. Therefore, the absorption spectrum could maintain its quality.

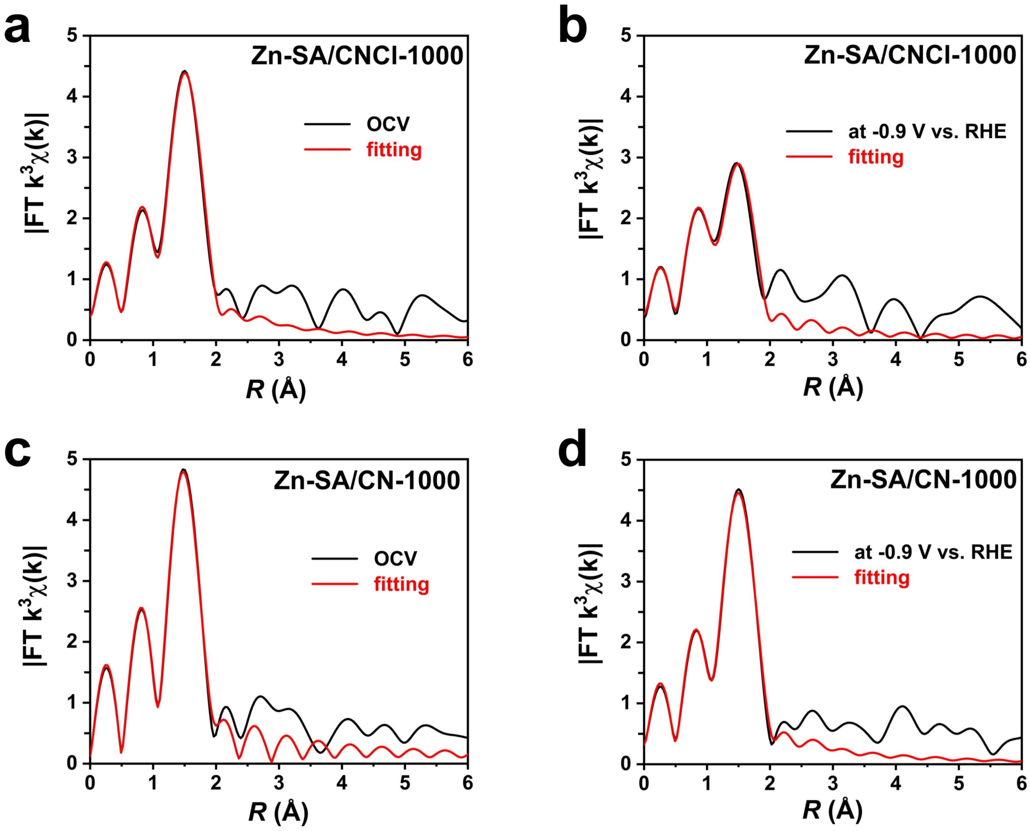

Supplementary Fig. 40. a, The fitting result of Zn-SA/CNCl-1000 in *R* space at OCV. **b**, The fitting result of Zn-SA/CNCl-1000 in *R* space at -0.9 V vs. RHE. **c**, The fitting result of Zn-SA/CN-1000 in *R* space at OCV. **d**, The fitting result of Zn-SA/CN-1000 in *R* space at -0.9 V vs. RHE. The potentials are provided without iR correction.

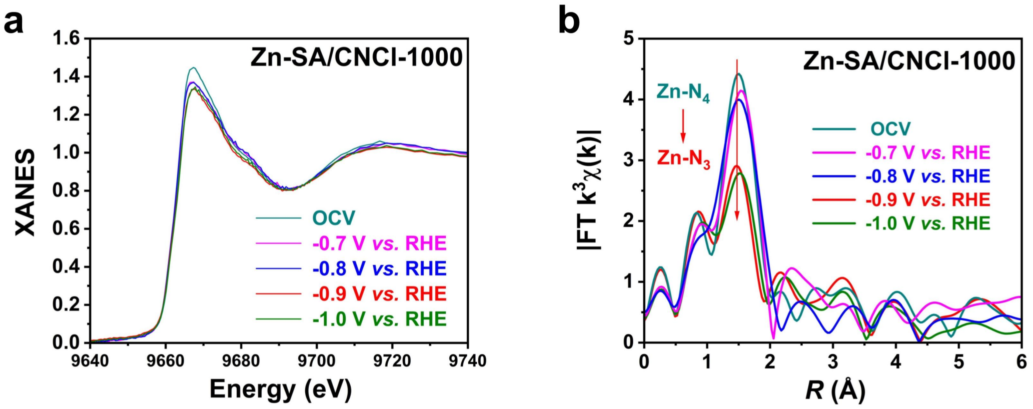

Supplementary Fig. 41. a, The XANES spectra for CO_2RR catalyzed by Zn-SA/CNCl-1000 at OCV, -0.7 V, -0.8 V, -0.9 V and -1.0 V *vs.* RHE. **b**, The corresponding FT-EXAFS spectra in *R* space at OCV, -0.7 V, -0.8 V, -0.9 V and -1.0 V *vs.* RHE. The potentials are provided without iR correction.

As shown in **Supplementary Fig. 41**, in-situ XANES and EXAFS measurements were carried out for CO_2RR catalyzed by Zn-SA/CNCl-1000 catalyst at OCV, -0.7 V, -0.8 V, -0.9 V and -1.0 V *vs.* RHE. As shown in **Supplementary Fig. 41a**, compared with the XANES curve at OCV, the intensities of XANES white-line (WL) at -0.7 V and -0.8 V *vs.* RHE had an obvious decline. The intensities of XANES white-line (WL) at -0.9 V and -1.0 V *vs.* RHE further decreased compared with those at -0.7 V and -0.8 V *vs.* RHE. The corresponding FT-EXAFS spectra in *R* space were shown in **Supplementary Fig. 41b**. As the applied potentials gradually decreased from OCV to -1.0 V *vs.* RHE, the intensities of main peak from Zn-N bond at around 1.5 Å gradually decreased correspondingly, revealing the gradual transformation from Zn-N_4 site into low-coordinated Zn-N_3 site. As shown in **Fig. 3b** and **Fig. 3c**, as the the applied potentials gradually decreased from -0.57 V to -0.93 V *vs.* RHE, both the J_{CO} and TOF values were remarkably increased, which was ascribed to the gradual transformation from Zn-N_4 site into low-coordinated Zn-N_3 site.

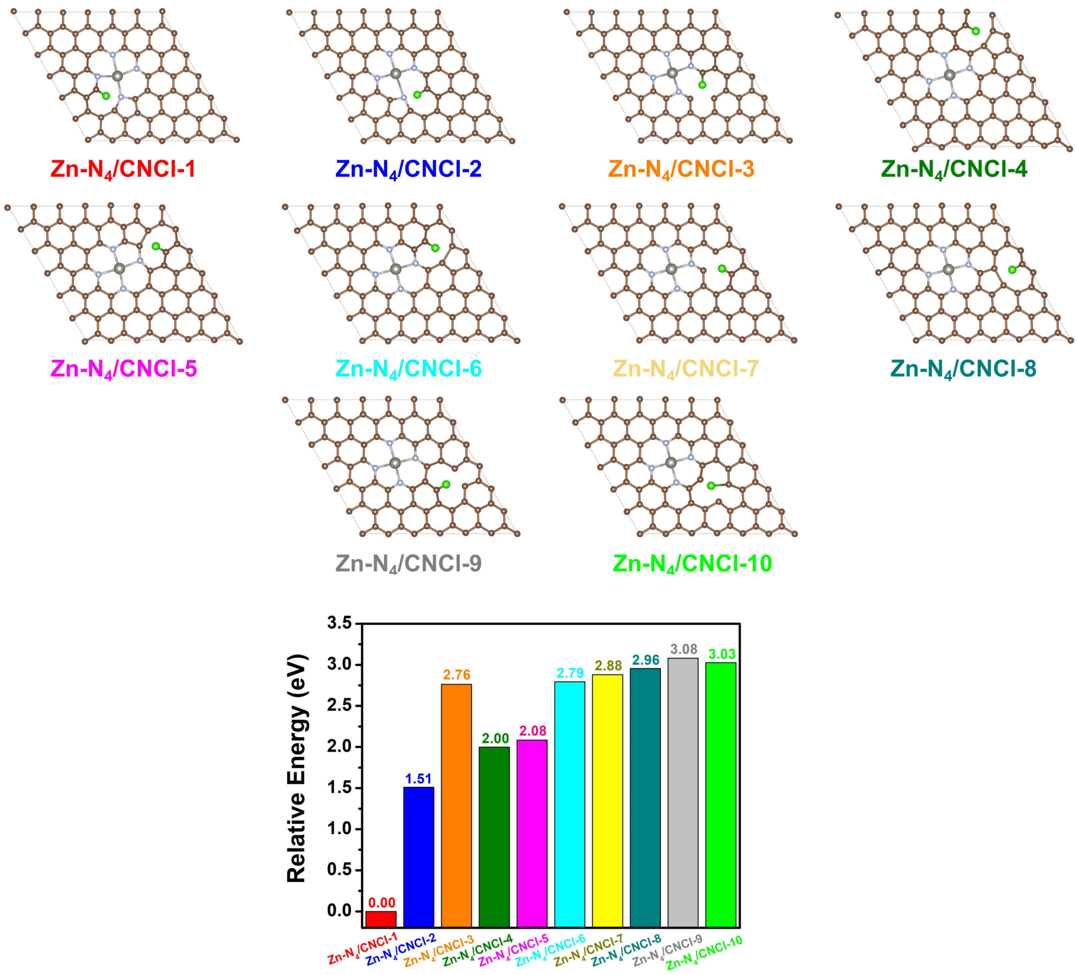

Supplementary Fig. 42. The optimized structures of ten possible Zn-N_4/CNCl models with C-Cl bond at different positions and the comparison of relative energies.

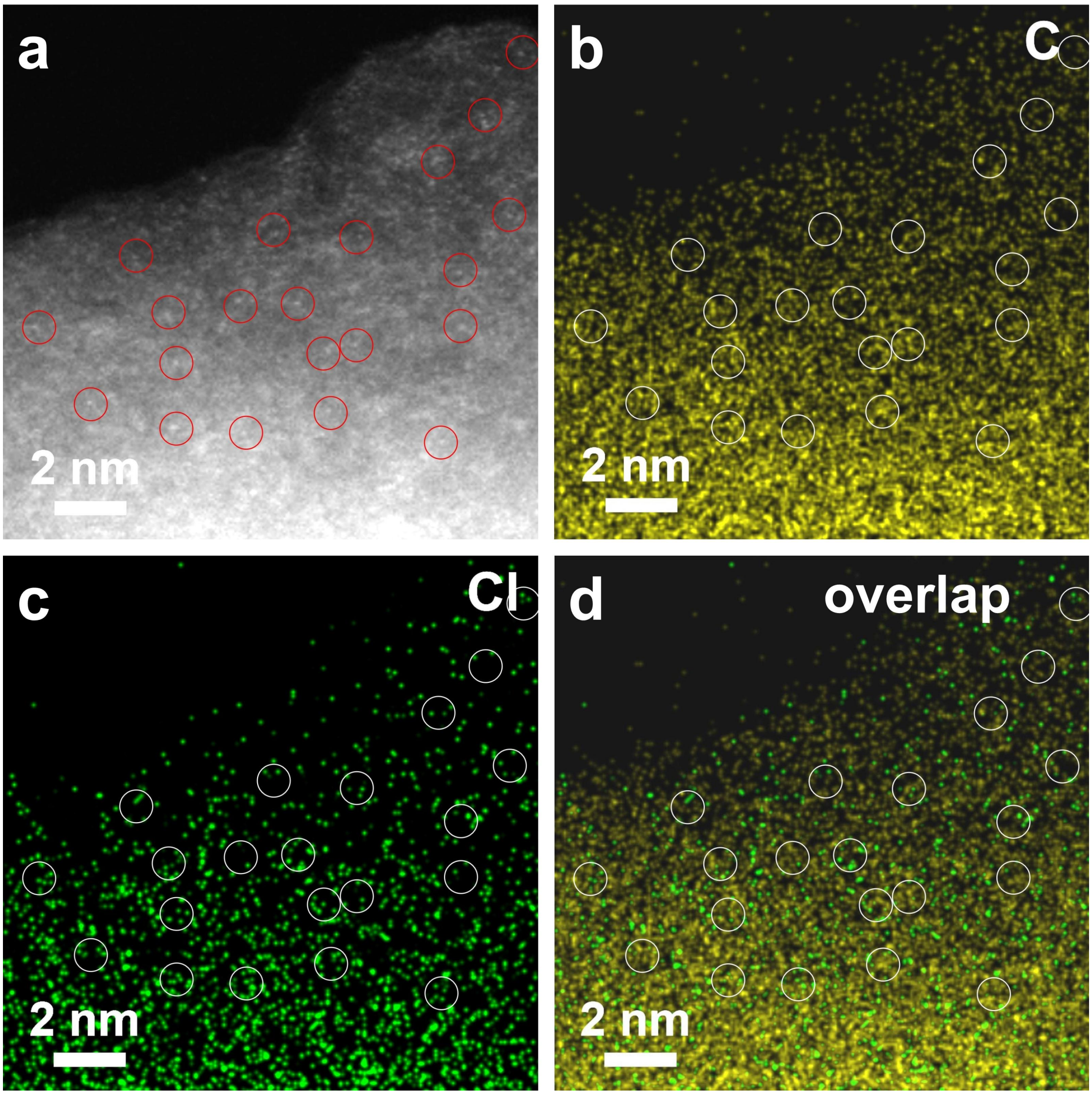

Supplementary Fig. 43. a, The AC-STEM image of Zn-SA/CNCl-1000. The isolated bright dots in Zn-SA/CNCl-1000 catalyst represent the Zn isolated single-atom sites, which are marked by red circles. **b-c**, The corresponding EDX spectroscopy elemental mapping results of C and Cl elements. **d**, The overlapping EDX spectroscopy elemental mapping results of C and Cl elements. The white circles in **Supplementary Fig. 43b-d** have the same locations as the red circles in **Supplementary Fig. 43a**, reflecting the locations of Zn isolated single-atom sites.

In order to prove the C-Cl bonds are indeed located in the vicinity of Zn species in Zn-SA/CNCl-1000, the AC-STEM measurement with EDX spectroscopy elemental mapping was carried out. As shown in **Supplementary Fig. 43a**, the isolated bright dots in Zn-SA/CNCl-1000 catalyst represented the Zn isolated single-atom sites,

which were marked by red circles. The corresponding EDX spectroscopy elemental mapping results of C and Cl elements were exhibited in **Supplementary Fig. 43b** and **Supplementary Fig. 43c**, respectively. The overlapping EDX spectroscopy elemental mapping results of C and Cl elements were exhibited in **Supplementary Fig. 43d**.

The C and Cl elements were homogeneously dispersed on the substrate of Zn-SA/CNCl-1000. The white circles in **Supplementary Fig. 43b-d** had the same locations as the red circles in **Supplementary Fig. 43a**, reflecting the locations of Zn isolated single-atom sites. As shown in **Supplementary Fig. 43d**, the C and Cl signal simultaneously appeared within each white circle, revealing that the C and Cl elements were indeed located in the vicinity of Zn species.

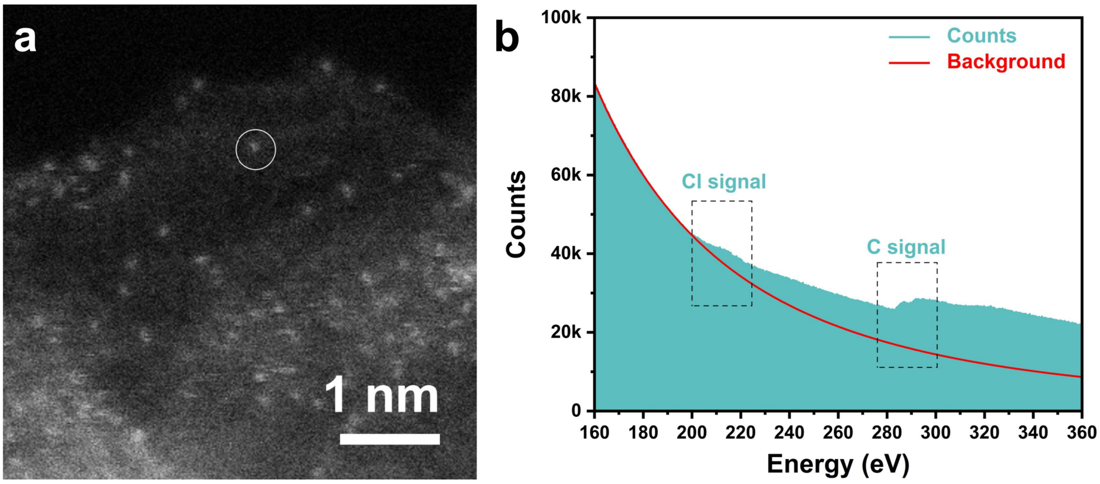

Supplementary Fig. 44. a, The AC-STEM image of Zn-SA/CNCl-1000. **b**, The EELS spectrum around the Zn isolated single-atom site, which is marked by the white circle in **Supplementary Fig. 44a**.

On the other hand, we also performed the AC-STEM measurement with electron energy loss spectroscopy (EELS). As shown in **Supplementary Fig. 44**, the EELS spectrum around the Zn isolated single-atom site confirmed the presence of C and Cl signals in the vicinity of Zn isolated single-atom site, also revealing the C and Cl elements were indeed located in the vicinity of Zn species.

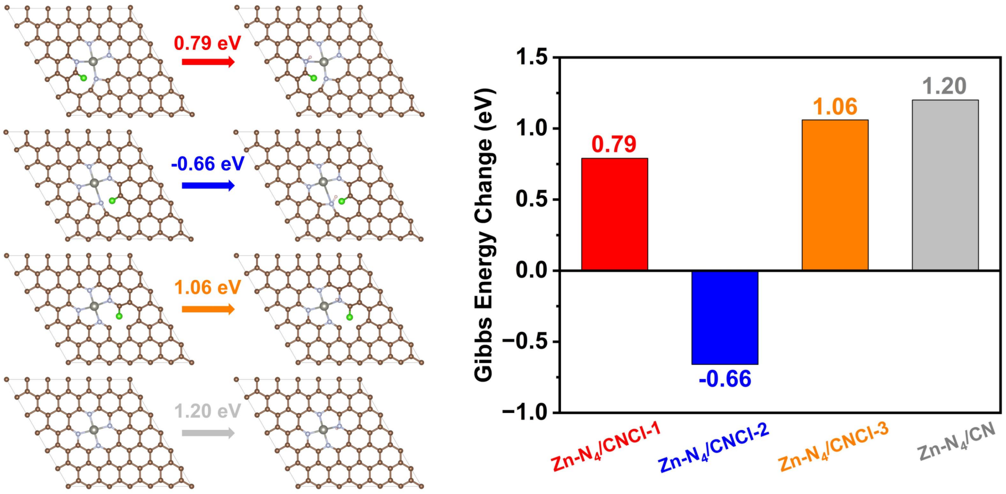

Supplementary Fig. 45. The Gibbs energy changes (ΔG) for the protonation of coordinated N atom on Zn-N_4/CNCl-1, Zn-N_4/CNCl-2, Zn-N_4/CNCl-3 and Zn-N_4/CN models and the corresponding optimized structures.

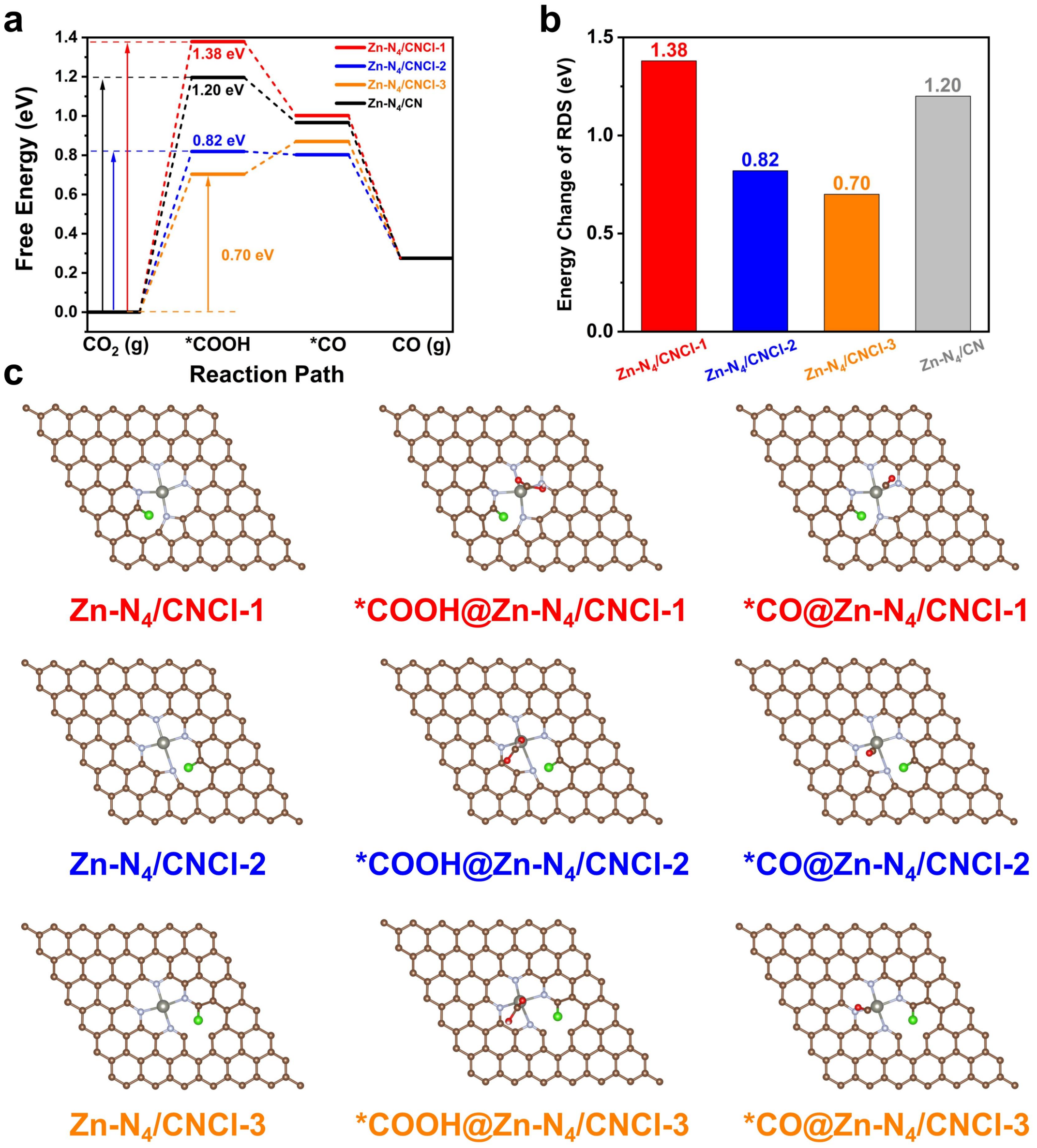

Supplementary Fig. 46. **a**, The energy changes for CO_2RR on Zn-N_4/CNCl-1, Zn-N_4/CNCl-2, Zn-N_4/CNCl-3 and Zn-N_4/CN models without the protonation of coordinated N atom. **b**. The comparison of energy changes for RDS. **c**, The optimized structures of *COOH and *CO on Zn-N_4/CNCl-1, Zn-N_4/CNCl-2 and Zn-N_4/CNCl-3 models. The direct CO_2RR pathways on Zn-N_4/CNCl-1, Zn-N_4/CNCl-2, Zn-N_4/CNCl-3 models were unable to induce the self-reconstruction of Zn-N_4 site into the low-coordinated Zn-N_3 catalytic sites.

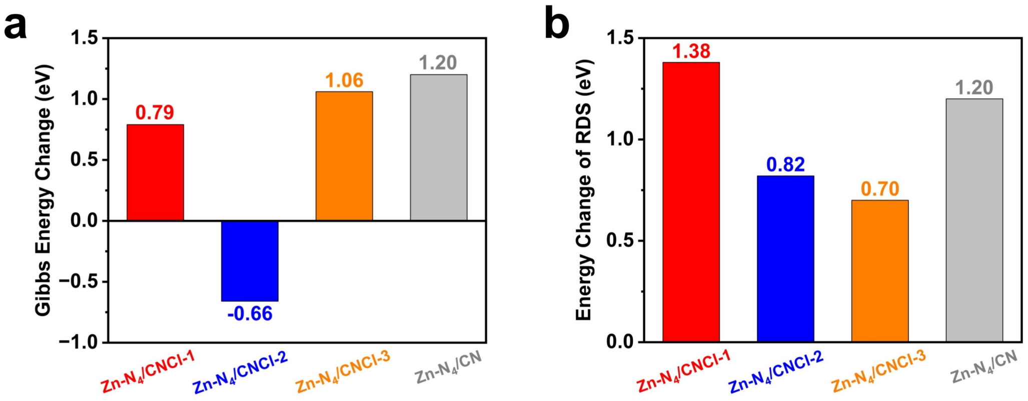

Supplementary Fig. 47. **a**, The comparison between energy changes of the protonation of coordinated N atom on Zn-N_4/CNCl and Zn-N_4/CN models. **b**, The comparison between the energy barriers of RDS for direct CO_2RR.

The energy barriers of RDS for direct CO_2RR on Zn-N_4/CNCl-1 and Zn-N_4/CNCl-2 models were 1.38 eV and 0.82 eV, respectively, much higher than their protonation of coordinated N atom (0.79 eV and -0.66 eV, respectively), indicating the protonation of coordinated N atom was easier to occur than direct CO_2RR on Zn-N_4/CNCl-1 and Zn-N_4/CNCl-2 models. For Zn-N_4/CNCl-3 model, the energy barrier of RDS for direct CO_2RR was 0.70 eV, lower than the protonation of coordinated N atom on Zn-N_4/CNCl-3 model. However, considering the Zn-N_4/CNCl-1 and Zn-N_4/CNCl-2 were more stable than Zn-N_4/CNCl-3, Zn-N_4/CNCl-1 and Zn-N_4/CNCl-2 as dominant models were more easier formed rather than Zn-N_4/CNCl-3 model. Besides, after the protonation of coordinated N atom on Zn-N_4/CNCl-3 model (Zn-N_4/CNCl-3(H)), the energy barrier of RDS for CO_2RR on Zn-N_4/CNCl-3(H) was 0.65 eV, lower than that of direct CO_2RR (0.70 eV) on Zn-N_4/CNCl-3 model. For Zn-N_4/CN model, both the energy barrier of RDS for direct CO_2RR and the protonation of coordinated N atom on Zn-N_4/CN model were 1.20 eV, indicating that there was no advantage for the protonation of coordinated N atom on Zn-N_4/CN model compared with direct CO_2RR pathway. Therefore, the protonation of coordinated N atom on Zn-N_4/CNCl models was more advantageous than their direct CO_2RR pathway.

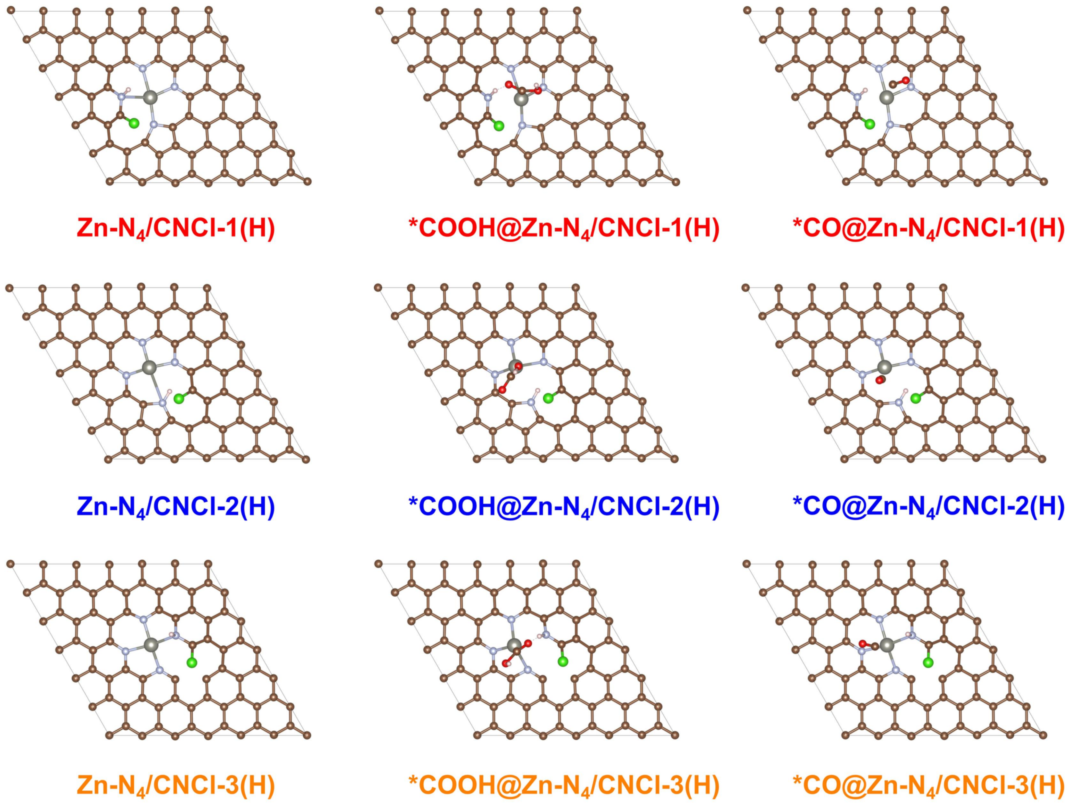

Supplementary Fig. 48. The optimized structures of *COOH and *CO on Zn-N$_4$/CNCl-1(H), Zn-N$_4$/CNCl-2(H) and Zn-N$_4$/CNCl-3(H) models.

Supplementary Table 1. The atomic ratios of C, N, Zn and Cl elements of Zn-SA/CNCl catalysts by XPS measurement.

Catalyst	Zn-SA/CNCl-850	Zn-SA/CNCl-920	Zn-SA/CNCl-1000
C (at%)	85.62	89.94	92.74
N (at%)	12.57	8.87	6.66
Zn (at%)	0.98	0.67	0.35
Cl (at%)	0.83	0.52	0.25

As shown in **Supplementary Table 1**, the atomic ratios of C, N, Zn and Cl elements of Zn-SA/CNCl catalysts are measured by XPS measurement. Although the atomic ratio of Cl element in Zn-SA/CNCl catalysts was much lower than those of C and N elements, the atomic ratio of Cl element in Zn-SA/CNCl catalysts was comparable to that of Zn element as catalytic sites. Therefore, the catalytic role of Cl element on Zn catalytic sites for CO_2RR can not be ignored.

Supplementary Table 2. The structural parameters extracted from the Zn K-edge for fitting Zn-SA/CNCl-850 with different models. (S_0^2 = 0.78)

Sample	Shell	N	R(Å)	σ^2(Å^2)	ΔE_0(eV)	R factor
Zn-SA/CNCl-850 (model 1)	Zn-N	4.11 ± 0.11	2.038 ± 0.011	0.0094 ± 0.0007	3.07 ± 0.28	0.001319
Zn-SA/CNCl-850 (model 2)	Zn-N	4.08 ± 0.16	2.027 ± 0.003	0.0099 ± 0.0011	0.98 ± 0.33	0.001805
	Zn-Cl	0.39 ± 0.10	2.217 ± 0.021	0.0194 ± 0.0050		
Zn-SA/CNCl-850 (model 3)	Zn-N1	4.09 ± 0.16	2.044 ± 0.015	0.0096 ± 0.0005	4.42 ± 0.37	0.001919
	Zn-N2	0.34 ± 0.12	2.214 ± 0.024	0.0155 ± 0.0062		

Supplementary Table 3. The structural parameters extracted from the Zn K-edge for fitting Zn-SA/CNCl-920 with different models. (S_0^2 = 0.78)

Sample	Shell	N	R(Å)	σ^2(Å^2)	ΔE_0(eV)	R factor
Zn-SA/CNCl-920 (model 1)	Zn-N	4.06 ± 0.12	2.025 ± 0.008	0.0092 ± 0.0005	1.74 ± 0.24	0.000804
Zn-SA/CNCl-920 (model 2)	Zn-N	4.03 ± 0.12	2.011 ± 0.012	0.0099 ± 0.0011	-0.56 ± 0.30	0.001445
	Zn-Cl	0.29 ± 0.05	2.218 ± 0.015	0.0120 ± 0.0050		
Zn-SA/CNCl-920 (model 3)	Zn-N1	4.04 ± 0.11	2.026 ± 0.011	0.0092 ± 0.0015	2.67 ± 0.50	0.001520
	Zn-N2	0.30 ± 0.13	2.217 ± 0.022	0.0094 ± 0.0015		

Supplementary Table 4. The structural parameters extracted from the Zn K-edge for fitting Zn-SA/CNCl-1000 with different models. (S_0^2 = 0.78)

Sample	Shell	N	R(Å)	σ^2(Å^2)	ΔE_0(eV)	R factor
Zn-SA/CNCl-1000 (model 1)	Zn-N	4.02 ± 0.14	2.045 ± 0.003	0.0098 ± 0.0005	4.22 ± 0.39	0.002235
Zn-SA/CNCl-1000 (model 2)	Zn-N	4.02 ± 0.14	2.045 ± 0.005	0.0098 ± 0.0021	4.32 ± 0.88	0.002278
	Zn-Cl	0.00 ± 0.17	2.219 ± 0.011	0.0113 ± 0.0056		

In the Zn-SA/CNCl catalysts, the N element is abundant while the Cl element is in trace amounts. Thus, the Zn elements in both Zn-SA/CNCl-850, Zn-SA/CNCl-920 and Zn-SA/CNCl-1000 mainly existed as Zn-N bond rather than Zn-Cl bond. Therefore, during the EXAFS fitting by model 2 with both Zn-N and Zn-Cl paths, the intensity of Zn-N bond is much stronger than that of the Zn-Cl bond. Thus, on the background of strong contribution of Zn-N bond, obtaining the accurate coordination number of Zn-Cl bond is challenging because the contribution of Zn-N bond can easily cover the contribution of Zn-Cl bond. Considering the strong contribution of Zn-N bond, it is difficult to judge whether the Zn-Cl bond exists in the Zn-SA/CNCl catalysts by only measuring the EXAFS results at Zn K-edge.

Supplementary Table 5. The structural parameters extracted from the Zn K-edge for fitting the first and second coordination shells of Zn atom in Zn-SA/CNCl catalysts. (S_0^2 = 0.78)

Sample	Shell	N	R(Å)	σ^2(Å^2)	ΔE_0(eV)	R factor
Zn-SA/CNCl-850	Zn-N	4.02 ± 0.08	2.034 ± 0.002	0.0092 ± 0.0004	2.49 ± 0.21	0.002692
	Zn-C-1	1.60 ± 0.46	2.899 ± 0.025	0.0127 ± 0.0037		
	Zn-C-2	4.41 ± 0.62	3.271 ± 0.013	0.0129 ± 0.0018		
Zn-SA/CNCl-920	Zn-N	4.01 ± 0.09	2.021 ± 0.003	0.0090 ± 0.0002	0.97 ± 0.19	0.001147
	Zn-C-1	1.21 ± 0.35	2.909 ± 0.020	0.0103 ± 0.0029		
	Zn-C-2	3.89 ± 0.44	3.262 ± 0.010	0.0109 ± 0.0013		
Zn-SA/CNCl-1000	Zn-N	3.98 ± 0.09	2.040 ± 0.002	0.0100 ± 0.0003	3.54 ± 0.16	0.001284
	Zn-C-1	1.69 ± 0.35	2.887 ± 0.015	0.0122 ± 0.0026		
	Zn-C-2	4.45 ± 0.47	3.278 ± 0.009	0.0125 ± 0.0013		

Supplementary Table 6. The structural parameters extracted from the Cl K-edge for fitting Zn-SA/CNCl samples.

Sample	Shell	N	R(Å)	$\sigma^2(10^{-3}Å^2)$	ΔE_0(eV)	R factor
Zn-SA/CNCl-850	Cl-C	0.47	1.67	2.12	-2.5	0.008
	Cl-Zn	0.55	2.38	1.51		
Zn-SA/CNCl-920	Cl-C	0.77	1.67	0.35	-4.4	0.017
	Cl-Zn	0.32	2.37	7.31		
Zn-SA/CNCl-1000	Cl-C	0.92	1.73	5.43	-0.4	0.025
	Cl-Zn	0.13	2.65	9.17		

Error bounds that characterize the structural parameters obtained by EXAFS spectroscopy were estimated as N ± 10%; R ± 1%; σ^2 ± 15%; ΔE_0 ± 20%.

Zn-SA/CNCl-850 (*k* range:3.0-10.0 $Å^{-1}$; *R* range: 1.0-3.0 Å)
Zn-SA/CNCl-920 (*k* range:3.0-10.0 $Å^{-1}$; *R* range: 1.0-3.0 Å)
Zn-SA/CNCl-1000 (*k* range:3.0-10.0 $Å^{-1}$; *R* range: 1.0-3.0 Å)

Supplementary Table 7. The structural parameters extracted from the Zn K-edge for fitting the first and second coordination shells of Zn atom in Zn-SA/CN-1000. (S_0^2 = 0.78)

Sample	Shell	N	R(Å)	σ^2(Å^2)	ΔE_0(eV)	R factor
Zn-SA/CN-1000	Zn-N	4.00 ± 0.09	2.001 ± 0.003	0.0081 ± 0.0003	-2.77 ± 0.31	0.000887
	Zn-C-1	0.85 ± 0.53	2.495 ± 0.023	0.0161 ± 0.0033		
	Zn-C-2	1.03 ± 0.69	3.259 ± 0.049	0.0164 ± 0.0052		

Supplementary Table 8. Comparison of catalytic performance for CO_2RR into CO in MEA full cells with Zn-SA/CNCl-1000 and other reported catalysts.

Catalyst	Anode electrolyte	Potential (V)	FE_{CO}	j_{CO} (mA/cm^2)	Reference
**Zn-SA/CNCl-1000**	**1M KOH**	**-0.93**	**97%**	**271.7**	**This work**
CoPc	1M KOH	-2.5	~95%	~175	*Science* **2019**, *365*, 367-369.
Au	Water	-2.65	> 85%	~300	*Energy Environ. Sci.* **2019**, *12*, 2455-2462.
Ni-N-C	1 M $KHCO_3$	-1.0	85%	~200	*Energy Environ. Sci.* **2019**, *12*, 640-647.
NiSA/PCFM	0.5 M $KHCO_3$	-1.0	88%	308.4	*Nat. Commun.* **2020**, *11*, 593-600.
Ag	Water with 1M CsOH activation	-3.0	~85%	~330	*Nat. Energy* **2021**, *6*, 439-448.
NiSA/NP	1 M KOH	-2.3	99%	310	*Angew. Chem. Int. Ed.* **2022**, *61*, e202203335.
Fe_1-NSC	1 M KOH	-0.35	98.8%	40	*Angew. Chem. Int. Ed.* **2022**, *61*, e202206233.

Supplementary Table 9. Structural parameters extracted from the Zn K-edge EXAFS fitting. (S_0^2 = 0.78)

Sample	Shell	N	R(Å)	σ^2(Å^2)	ΔE_0(eV)	R factor
Zn foil	Zn-Zn^1	6*	2.644 ± 0.005	0.0096 ± 0.0004	3.64 ± 0.66	0.0007218
	Zn-Zn^2	6*	2.766 ± 0.007	0.0185 ± 0.0023		
Zn-SA/CNCl-1000 (CO_2RR@OCV)	Zn-N	4.04 ± 0.17	2.030 ± 0.003	0.0094 ± 0.0006	3.69 ± 0.42	0.0021748
Zn-SA/CNCl-1000 (CO_2RR@-0.9V)	Zn-N	3.44 ± 0.35	2.008 ± 0.007	0.0134 ± 0.0014	2.24 ± 0.89	0.0052816
Zn-SA/CN-1000 (CO_2RR@OCV)	Zn-N	4.31 ± 0.11	2.024 ± 0.002	0.0100 ± 0.0003	2.43 ± 0.23	0.0005161
Zn-SA/CN-1000 (CO_2RR@-0.9V)	Zn-N	4.07 ± 0.09	2.021 ± 0.002	0.0093 ± 0.0003	2.70 ± 0.22	0.0005811
